# Distance decay 2.0 – A global synthesis of taxonomic and functional turnover in ecological communities

**DOI:** 10.1111/geb.13513

**Published:** 2022-05-12

**Authors:** Caio Graco‐Roza, Sonja Aarnio, Nerea Abrego, Alicia T. R. Acosta, Janne Alahuhta, Jan Altman, Claudia Angiolini, Jukka Aroviita, Fabio Attorre, Lars Baastrup‐Spohr, José J. Barrera‐Alba, Jonathan Belmaker, Idoia Biurrun, Gianmaria Bonari, Helge Bruelheide, Sabina Burrascano, Marta Carboni, Pedro Cardoso, José C. Carvalho, Giuseppe Castaldelli, Morten Christensen, Gilsineia Correa, Iwona Dembicz, Jürgen Dengler, Jiri Dolezal, Patricia Domingos, Tibor Erös, Carlos E. L. Ferreira, Goffredo Filibeck, Sergio R. Floeter, Alan M. Friedlander, Johanna Gammal, Anna Gavioli, Martin M. Gossner, Itai Granot, Riccardo Guarino, Camilla Gustafsson, Brian Hayden, Siwen He, Jacob Heilmann‐Clausen, Jani Heino, John T. Hunter, Vera L. M. Huszar, Monika Janišová, Jenny Jyrkänkallio‐Mikkola, Kimmo K. Kahilainen, Julia Kemppinen, Łukasz Kozub, Carla Kruk, Michel Kulbiki, Anna Kuzemko, Peter Christiaan le Roux, Aleksi Lehikoinen, Domênica Teixeira de Lima, Angel Lopez‐Urrutia, Balázs A. Lukács, Miska Luoto, Stefano Mammola, Marcelo M. Marinho, Luciana S. Menezes, Marco Milardi, Marcela Miranda, Gleyci A. O. Moser, Joerg Mueller, Pekka Niittynen, Alf Norkko, Arkadiusz Nowak, Jean P. Ometto, Otso Ovaskainen, Gerhard E. Overbeck, Felipe S. Pacheco, Virpi Pajunen, Salza Palpurina, Félix Picazo, Juan A. C. Prieto, Iván F. Rodil, Francesco M. Sabatini, Shira Salingré, Michele De Sanctis, Angel M. Segura, Lucia H. S. da Silva, Zora D. Stevanovic, Grzegorz Swacha, Anette Teittinen, Kimmo T. Tolonen, Ioannis Tsiripidis, Leena Virta, Beixin Wang, Jianjun Wang, Wolfgang Weisser, Yuan Xu, Janne Soininen

**Affiliations:** ^1^ Department of Geosciences and Geography University of Helsinki Helsinki Finland; ^2^ Laboratory of Ecology and Physiology of Phytoplankton Department of Plant Biology State University of Rio de Janeiro Rio de Janeiro RJ Brazil; ^3^ Department of Agricultural Sciences University of Helsinki Helsinki Finland; ^4^ Department of Biological and Environmental Science University of Jyväskylä Jyväskylä Finland; ^5^ Department of Science University of Roma Tre Rome Italy; ^6^ Geography Research Unit University of Oulu Oulu Finland; ^7^ Freshwater Centre Finnish Environment Institute Oulu Finland; ^8^ Institute of Botany The Czech Academy of Sciences Průhonice Czech Republic; ^9^ Faculty of Forestry and Wood Sciences Czech University of Life Sciences Prague Czech Republic; ^10^ Department of Life Sciences University of Siena Siena Italy; ^11^ Department of Environmental Biology Sapienza University of Rome Rome Italy; ^12^ Freshwater Biological Laboratory Department of Biology University of Copenhagen Universitetsparken København Ø Denmark; ^13^ Departamento de Ciências do Mar Instituto do Mar Universidade Federal de São Paulo. R. Carvalho de Mendonça Santos SP Brazil; ^14^ George S. Wise Faculty of Life Sciences School of Zoology Tel Aviv University Tel Aviv Israel; ^15^ Steinhardt Museum of Natural History Tel Aviv University Tel Aviv Israel; ^16^ Department of Plant Biology and Ecology University of the Basque Country UPV/EHU Bilbao Spain; ^17^ Faculty of Science and Technology Free University of Bozen‐Bolzano Bozen‐Bolzano Italy; ^18^ Institute for Biology/Geobotany and Botanical Garden Martin Luther University Halle‐Wittenberg Halle Germany; ^19^ German Centre for Integrative Biodiversity Research (iDiv) Halle‐Jena‐Leipzig Leipzig Germany; ^20^ Laboratory for Integrative Biodiversity Research (LIBRe) Finnish Museum of Natural History Luomus University of Helsinki Helsinki Finland; ^21^ Department of Biology CBMA—Centre for Molecular and Environmental Biology University of Minho Braga Portugal; ^22^ Department of Life Sciences and Biotechnology University of Ferrara Ferrara Italy; ^23^ Morten Chr. Consult Sorø Denmark; ^24^ Department of Ecology and Environmental Conservation Faculty of Biology Institute of Environmental Biology University of Warsaw Warsaw Poland; ^25^ Vegetation Ecology Institute of Natural Resource Sciences (IUNR) Zurich University of Applied Sciences (ZHAW) Wädenswil Switzerland; ^26^ Plant Ecology Bayreuth Center of Ecology and Environmental Research (BayCEER) Bayreuth Germany; ^27^ Department of Botany Faculty of Science University of South Bohemia České Budějovice Czech Republic; ^28^ Laboratory of Phycology and Environmental Education Department of Plant Biology State University of Rio de Janeiro Rio de Janeiro RJ Brazil; ^29^ Balaton Limnological Research Institute ELKH Tihany Hungary; ^30^ Reef Systems Ecology and Conservation Lab Departamento de Biologia Marinha Universidade Federal Fluminense Niterói RJ Brazil; ^31^ Department of Agricultural and Forest Sciences (DAFNE) University of Tuscia Viterbo Italy; ^32^ Marine Macroecology and Biogeography Lab Departamento de Ecologia e Zoologia CCB Universidade Federal de Santa Catarina Florianopolis SC Brazil; ^33^ Hawai’i Institute of Marine Biology University of Hawai’i Kaneohe Hawaii USA; ^34^ Pristine Seas National Geographic Society Washington District of Columbia USA; ^35^ Tvärminne Zoological Station University of Helsinki Hanko Finland; ^36^ Forest Entomology Swiss Federal Research Institute WSL Birmensdorf Switzerland; ^37^ Department of Environmental Systems Science Institute of Terrestrial Ecosystems ETH Zurich Zurich Switzerland; ^38^ Department STEBICEF – Botanical Unit University of Palermo Palermo Italy; ^39^ Canadian Rivers Institute Biology Department University of New Brunswick Fredericton New Brunswick Canada; ^40^ Department of Entomology College of Plant Protection Nanjing Agricultural University Nanjing China; ^41^ Centre for Macroecology, Evolution and Climate University of Copenhagen Copenhagen Ø Denmark; ^42^ School of Rural and Environmental Sciences University of New England Armidale New South Wales Australia; ^43^ Phycology Laboratory Botany Department National Museum Federal University of Rio de Janeiro Rio de Janeiro RJ Brasil; ^44^ Plant Science and Biodiversity Center Institute of Botany Slovak Academy of Sciences Banská Bystrica Slovakia; ^45^ Lammi Biological Station University of Helsinki Lammi Finland; ^46^ Sección Limnología IECA Facultad de Ciencias Universidad de la República Montevideo Uruguay; ^47^ Ecología Funcional de Sistemas Acuáticos CURE‐Rocha Universidad de la República Montevideo Uruguay; ^48^ Laboratoire d’Excellence Labex Corail UMR IRD‐UR‐CNRS ENTROPIE IRD (Institut de Recherche pour le Développement) Université de Perpignan Perpignan Cedex France; ^49^ National Academy of Sciences of Ukraine M.G. Kholodny Institute of Botany Kyiv Ukraine; ^50^ Department of Botany and Zoology Masaryk University Brno Czech Republic; ^51^ Department of Plant and Soil Sciences University of Pretoria Pretoria South Africa; ^52^ Finnish Museum of Natural History University of Helsinki Helsinki Finland; ^53^ Departamento de Oceanografia Biológica (DOB) Faculdade de Oceanografia Universidade do Estado do Rio de Janeiro (UERJ) Rio de Janeiro RJ Brasil; ^54^ Centro Oceanográfico de Gijón Instituto Español de Oceanografía (IEO) Gijón Asturias Spain; ^55^ Centre for Ecological Research Wetland Ecology Research Group Debrecen Hungary; ^56^ National Research Council (CNR) Molecular Ecology Group (MEG) Water Research Institute (IRSA) Pallanza Italy; ^57^ Laboratory of Grassland Vegetation Universidade Federal do Rio Grande do Sul Porto Alegre RS Brazil; ^58^ Fisheries New Zealand Tini a Tangaroa Ministry for Primary Industries Wellington New Zealand; ^59^ Earth System Science Center CCST/INPE National Institute for Space Research São José dos Campos SP Brazil; ^60^ Institute for Biochemistry and Biology University of Potsdam Potsdam Germany; ^61^ Department of Nature Conservation Heinz Sielmann Foundation Wustermark Germany; ^62^ Baltic Sea Centre Stockholm University Stockholm Sweden; ^63^ Center for Biological Diversity Conservation in Powsin Polish Academy of Sciences Botanical Garden Warsaw Poland; ^64^ Institute of Biology University of Opole Opole Poland; ^65^ Organismal and Evolutionary Biology Research Programme Faculty of Biological and Environmental Sciences University of Helsinki Helsinki Finland; ^66^ Department of Biology Centre for Biodiversity Dynamics Norwegian University of Science and Technology Trondheim Norway; ^67^ Department of Botany Universidade Federal do Rio Grande do Sul Alegre RS Brazil; ^68^ National Museum of Natural History Bulgarian Academy of Sciences Sofia Bulgaria; ^69^ Department of Ecology/Research Unit Modeling Nature (MNat) Faculty of Sciences University of Granada Granada Spain; ^70^ State Key Laboratory of Lake Science and Environment Nanjing Institute of Geography and Limnology Chinese Academy of Sciences Nanjing China; ^71^ Department of Biology International Campus of Excellence of the Sea (CEIMAR) INMAR University of Cadiz Puerto Real (Cádiz) Spain; ^72^ BIOME Lab Department of Biological, Geological and Environmental Sciences (BiGeA) Alma Mater Studiorum University of Bologna Bologna Italy; ^73^ Department of Environmental Biology University Sapienza of Rome Rome Italy; ^74^ Modelización y Análisis de Recursos Naturales CURE‐Rocha Universidad de la República Montevideo Uruguay; ^75^ Phycology Laboratory Botany Department National Museum Federal University of Rio de Janeiro Rio de Janeiro RJ Brazil; ^76^ Department of Agrobotany Faculty of Agriculture University of Belgrade Belgrade‐Zemun Serbia; ^77^ Botanical Garden University of Wrocław Wrocław Poland; ^78^ Freshwater Centre Finnish Environment Institute Jyväskylä Office University of Jyväskylä Jyväskylä Finland; ^79^ Department of Botany School of Biology Aristotle University of Thessaloniki Thessaloniki Greece; ^80^ Terrestrial Ecology Research Group Department of Ecology and Ecosystem Management Technical University of Munich Freising Germany; ^81^ State Key Laboratory of Estuarine and Coastal Research East China Normal University Shanghai China

**Keywords:** β‐diversity, biogeography, environmental gradient, spatial distance, trait

## Abstract

**Aim:**

Understanding the variation in community composition and species abundances (i.e., β‐diversity) is at the heart of community ecology. A common approach to examine β‐diversity is to evaluate directional variation in community composition by measuring the decay in the similarity among pairs of communities along spatial or environmental distance. We provide the first global synthesis of taxonomic and functional distance decay along spatial and environmental distance by analysing 148 datasets comprising different types of organisms and environments.

**Location:**

Global.

**Time period:**

1990 to present.

**Major taxa studied:**

From diatoms to mammals.

**Method:**

We measured the strength of the decay using ranked Mantel tests (Mantel *r*) and the rate of distance decay as the slope of an exponential fit using generalized linear models. We used null models to test whether functional similarity decays faster or slower than expected given the taxonomic decay along the spatial and environmental distance. We also unveiled the factors driving the rate of decay across the datasets, including latitude, spatial extent, realm and organismal features.

**Results:**

Taxonomic distance decay was stronger than functional distance decay along both spatial and environmental distance. Functional distance decay was random given the taxonomic distance decay. The rate of taxonomic and functional spatial distance decay was fastest in the datasets from mid‐latitudes. Overall, datasets covering larger spatial extents showed a lower rate of decay along spatial distance but a higher rate of decay along environmental distance. Marine ecosystems had the slowest rate of decay along environmental distances.

**Main conclusions:**

In general, taxonomic distance decay is a useful tool for biogeographical research because it reflects dispersal‐related factors in addition to species responses to climatic and environmental variables. Moreover, functional distance decay might be a cost‐effective option for investigating community changes in heterogeneous environments.

## 1 INTRODUCTION

Biodiversity on Earth is shrinking (IPBES, 2019). Understanding its distribution is therefore paramount to inform conservation efforts and to evaluate the links between biodiversity, ecosystem functioning, ecosystem services and human well‐being (Cardinale et al., 2012). The variation in the occurrence and abundance of species in space and time (i.e., β‐diversity) is at the heart of community ecology and biogeography because it provides a direct link between local (α) and regional (γ) diversity (Mori et al., 2018). Moreover, β‐diversity has become an essential currency in spatial (Kraft et al., 2011) and temporal (Blowes et al., 2019) comparisons of biodiversity patterns and their underlying drivers. The β‐diversity is also informative in the context of biodiversity conservation and practical management decisions in rapidly changing environments (Gossner et al., 2016).

A common approach to examine spatial β‐diversity is to consider directional turnover in community composition with distance (i.e., distance decay) (Anderson et al., 2011; Nekola & White, 1999). The similarity among the pairs of biological communities typically decreases (“decays”) with increasing spatial or environmental distance (Nekola & White, 1999). This pattern stems mainly from dispersal limitation [related to physical barriers and dispersal ability (Hubbell, 2001)] and species‐specific responses to spatially structured environmental variation [related to environmental filters and evolutionary processes (Cottenie, 2005)] and is well documented in observational (Astorga et al., 2012) and theoretical studies (Morlon et al., 2008) and in meta‐analyses (Soininen et al., 2007). Although the patterns and drivers of taxonomic β‐diversity are relatively well studied in the biogeographical literature, whether the same patterns occur for functional β‐diversity is much less understood (Villéger et al., 2012).

Understanding functional diversity relies on trait‐based approaches, which are built on the idea that the environment selects species based on their ecological requirements and that functional traits capture these requirements better than species identity (McGill et al., 2006). Thus, a trait‐based approach should reflect the functional response of biotic communities to environmental gradients better than an approach based only on the taxonomic identities of species and should predict better how biotic communities respond to environmental changes (Mouillot et al., 2013). Functional diversity has been investigated widely at the α‐diversity level (Buisson et al., 2013; Villéger et al., 2008), but our understanding of functional β‐diversity is much more limited and fragmented (Heino & Tolonen, 2017; Penone et al., 2016; Villéger et al., 2013). Comparing the patterns of functional and taxonomic β‐diversity across different biotic groups, ecosystems and geographical contexts has the potential to contribute greatly to a mechanistic understanding of the drivers behind the spatial variation in ecosystem functionality and shed further light on how environmental change might affect ecological communities.

**FIGURE 1:**
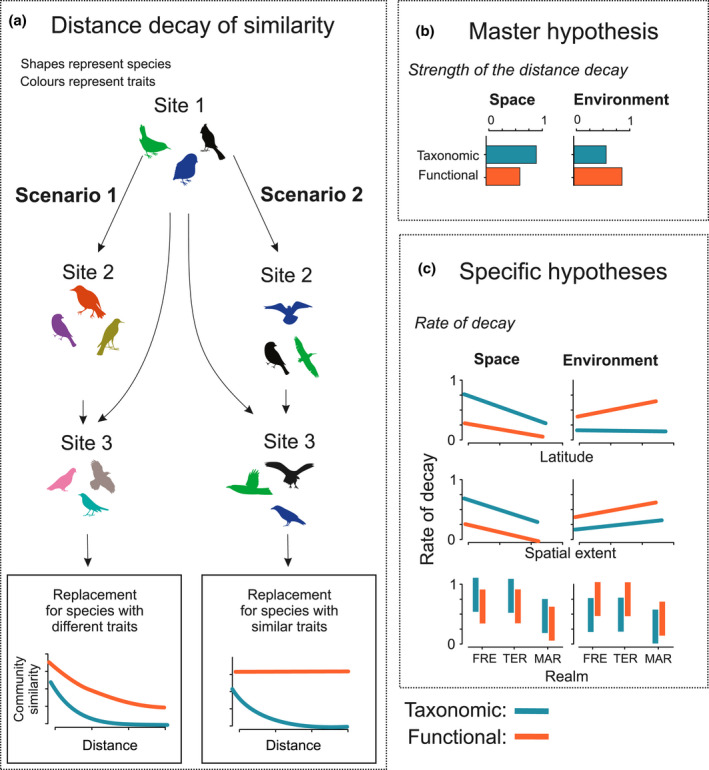
(a) Taxonomic and functional distance decay. Two scenarios of distance decay of taxonomic and functional similarities along spatial and environmental distance. In scenario 1 (for simplicity, we consider here replacement only), the replacement occurs among species that have different traits (i.e., colours), which leads to both taxonomic and functional distance decay. In scenario 2, the replacement occurs among species that have similar traits, which leads to zero functional distance decay measured by the slope. (b) Master hypothesis: spatial distance decay is stronger for taxonomic similarities than for functional similarities, whereas environmental distance decay is stronger for functional similarities. (c) Specific hypotheses (higher values indicate steeper slopes) across datasets. For latitude, spatial distance decay is flatter in the datasets from higher latitude and, more notably, for taxonomic similarities than for functional similarities. Environmental distance decay is steeper in datasets from higher latitude for functional similarities, whereas it does not vary notably with latitude for taxonomic similarities. For spatial extent, both taxonomic and functional spatial distance decay are flatter in the datasets covering a larger spatial extent, whereas environmental distance decay is steeper in datasets covering a larger extent. For realm, marine ecosystems show flatter spatial and environmental distance decay than terrestrial and freshwater systems. Abbreviations: FRE = freshwater systems; MAR = marine systems; TER = terrestrial systems

Several ecological processes can be inferred from the correlation between taxonomic and functional similarity. For example, a coupling of taxonomic and functional distance decay might indicate that species from the regional pool have equal probabilities of reaching all sites, but local communities are assembled based on local habitat constraints on organisms present at each site (Sokol et al., 2011). This generates functional clustering (i.e., trait variability is smaller than expected given the taxonomic composition) at the site level, but overdispersion (i.e., trait variability is larger than expected given the taxonomic composition) at the regional level. This phenomenon has been observed, for example, for specific leaf area of tree communities along an elevational gradient (Swenson et al., 2011). However, high taxonomic β‐diversity does not always mean high functional β‐diversity (Leibold & McPeek, 2006; Mouillot et al., 2013) (Figure 1a). In fact, a functional decay stronger than expected given the taxonomic decay might occur if the species in the two communities are functionally more divergent than expected given the species pool. In contrast, a weaker functional decay than expected given the taxonomic decay might occur if local communities under same habitat constraints are subsamples of multiple regional pools with different species composition. Therefore, the most pressing question is whether functional similarity decays typically faster or slower along environmental gradients than expected given the taxonomic decay, as suggested by some earlier studies (Carvalho et al., 2020; Sokol et al., 2011; Swenson et al., 2011).

### 1.1 Hypotheses

Following the first comprehensive distance decay meta‐analysis (Soininen, McDonald, et al., 2007), our understanding of community turnover along spatial and environmental gradients has increased notably. Here, based on existing ecological literature and theory, and as an initial step towards synthesizing knowledge, we tested four hypotheses concerning the differences between taxonomic and functional distance decay along the spatial and environmental distance. The master hypothesis (H_1a_) is that the distance decay along spatial gradients is stronger for taxonomic similarity than for functional similarity. This is because spatial factors relate more to taxonomic than functional composition owing to dispersal processes, dispersal history and speciation (Soininen et al., 2016). Such a hypothesis should be valid when functional traits do not comprise dispersal‐related traits. In contrast (H_1b_), distance decay along environmental gradients is stronger for functional similarity than for taxonomic similarity because functional composition should respond more strongly to environmental variation (Meynard et al., 2011; Soininen et al., 2016) (Figure 1b).

#### 1.1.1 Latitudinal gradients

We also generalize the effects of major geographical and environmental factors in the three hypotheses that are tested across the datasets. For example, latitudinal effect has been recognized as a relevant factor in meta‐analyses (Soininen et al., 2007) and case studies (Qian et al., 2009), and these studies suggest that β‐diversity should decrease with increasing latitude (Figure 1c). This is indicated by the faster latitudinal decline in γ‐diversity than in α‐diversity (Hillebrand, 2004; Soininen, 2010) and the decrease in slopes of the species–area relationships (proxy for turnover) with latitude (Drakare et al., 2005). Moreover, Rapoport's rule (Stevens, 1989) postulates that species range sizes are larger at high latitudes, leading to lower β‐diversity (but see Rohde, 1996). Therefore, we hypothesize (H_2a_) that the rate of taxonomic distance decay along spatial gradients is generally slower in the datasets that originate from higher latitudes. In contrast, functional distance decay along a spatial gradient might be faster in the datasets from higher latitudes because large‐scale environmental heterogeneity tends to increase towards the poles (Soininen & Hillebrand, 2007; Soininen, McDonald, et al., 2007; Terborgh, 1973). Thus, environmental filtering becomes stronger with increasing latitude (Jarzyna et al., 2021; Lamanna et al., 2014), leading to functionally clustered communities locally that become increasingly overdispersed along the regional environmental gradient. This would result in a faster rate of functional distance decay along environmental gradients at higher latitudes (H_2b_). An alternative hypothesis is that extreme climatic conditions at high latitudes decrease functional diversity because abiotic filtering limits the number of possible ecological strategies found in a biotic community (Cornwell & Ackerly, 2009), resulting in a relatively slow rate of functional distance decay.

#### 1.1.2 Spatial extent

Distance decay is also likely to be affected by the spatial extent of a given study (Nekola & McGill, 2014; Steinbauer et al., 2012). It has been shown that distance decay has a power‐law shape at spatial extents that do not exceed regional species pools and an exponential shape when extent encompasses multiple species pools (Nekola & McGill, 2014). This suggests that the slope of the relationship becomes flatter with increasing spatial extent (Soininen, McDonald, et al., 2007), mainly because regional species diversity is limited with a certain upper boundary (Triantis et al., 2011). Furthermore, environmental heterogeneity affects the diversity of species (Pianka, 1966) and functional traits at a regional level (Questad & Foster, 2008), but such effects are likely to be scale dependent (Gazol et al., 2013; Laanisto et al., 2012). To summarize, we hypothesize (H_3a_) that the rate of distance decay along spatial gradients is generally slower in the datasets covering larger spatial extent. In contrast (H_3b_), the rate of distance decay along environmental gradients is generally faster when the spatial extent is larger, especially for functional similarities.

#### 1.1.3 Realms

We also expect the patterns of distance decay to vary among realms. In general, marine ecosystems are environmentally more homogeneous than terrestrial or freshwater ecosystems, at least in the open ocean (Clarke, 1992), and typically show weaker dispersal barriers than terrestrial or freshwater ecosystems (Bierne et al., 2003; Cornell & Harrison, 2014). Therefore, we hypothesize (H_4_) that the datasets from marine ecosystems generally have slower rates of taxonomic and functional distance decay than the other ecosystems.

**FIGURE 2:**
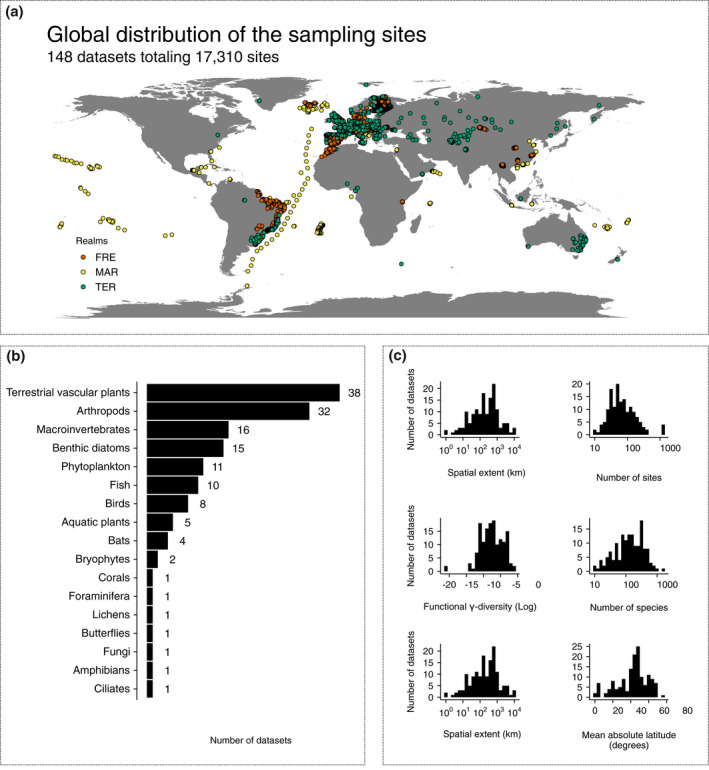
Study design highlighting (a) a map of the study sites coloured according to the realms (FRE = freshwater; MAR = marine; TER = terrestrial); (b) the number of datasets for major biotic groups; and (c) the distribution of the datasets with respect to spatial extent, number of study sites, functional γ‐diversity (log_10_ hypervolume SD^3^), taxonomic γ‐diversity (number of species), number of environmental variables and latitude

Here, we tested these hypotheses using datasets that cover a wide range of biotic groups, from unicellular diatoms to vascular plants, fungi, invertebrates, fish, birds, amphibians and mammals, and that originate from marine, terrestrial and freshwater ecosystems spanning broad latitudinal gradients (Figure 2). To account for major biological differences in biotic groups, we also investigated whether distance decay varied among different‐sized taxa or among taxa with different dispersal modes (Bie et al., 2012; Jenkins et al., 2007). By using such a comprehensive, multi‐realm and multi‐taxon dataset, we explore patterns at a more general level compared with case studies that have examined both taxonomic and functional β‐diversity but considered only a single or few biotic groups.

## 2 MATERIAL AND METHODS

### 2.1 Data collection

**FIGURE 3:**
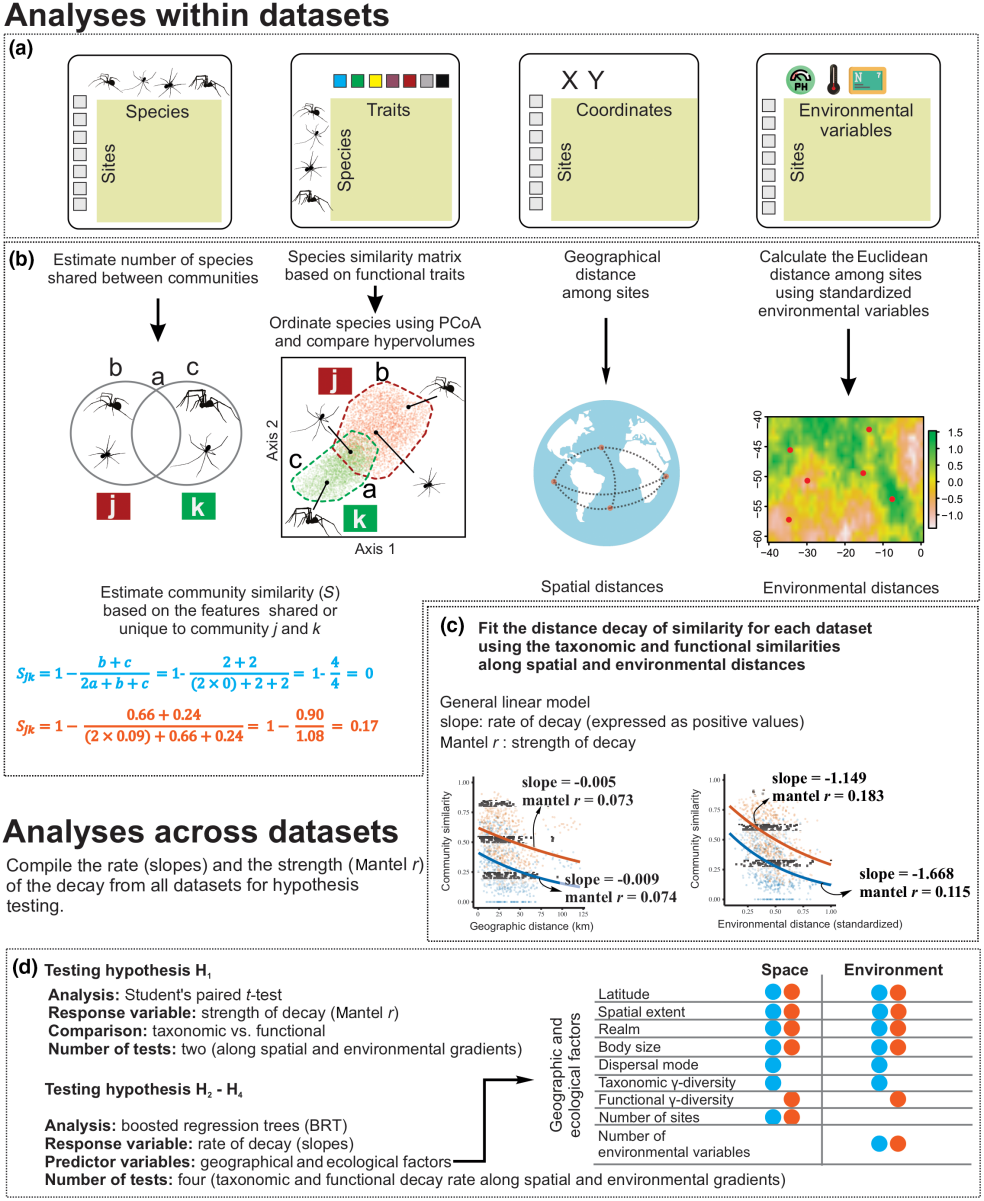
The analytical framework described in a stepwise manner: (a–c) hierarchical description of the methods performed at dataset level, including the estimation of similarities and distance in addition to the distance decay models of each dataset; and (d) description of the tests performed after the compilation of the metrics from all datasets. (a) The four objects used in the analyses: a species‐by‐traits table, a sites‐by‐species matrix, a sites‐by‐coordinates table and a sites‐by‐environment table. (b) The calculation of taxonomic and functional similarities and of spatial and environmental distance. In the first example, only species identities are considered, and because sites *k* and *k* do not share any species, community similarity (blue) equals zero. In the second example, the functional traits of species are considered, and community similarity (orange) is higher than zero. The third example shows how spatial distance was calculated as the geographical distance between pairs of sites using spatial coordinates. The fourth example illustrates how sites far from each other may show similar environmental conditions and therefore small environmental distance. Environmental distance was calculated as the Euclidean distance between pairs of sites considering the standardized environmental variables. (c) Illustration of the metrics extracted to study the distance decay across datasets. The strength of distance decay was measured from Mantel tests using Spearman correlations (Mantel *r*), and the rate of decay was measured as the slopes of generalized linear models following a quasibinomial family with a log link. The models were built separately for each response variable (taxonomic or functional similarity) and explanatory variables (spatial or environmental distance), totalling four Mantel *r* values and four slopes. Also, the data of marine fish from the Mediterranean Sea are shown as an example in which the distance decay of similarity along environmental distance is stronger (higher Mantel *r*) for functional similarity than for taxonomic similarity, irrespective of the rate of decay (slope). (d) Description of the analyses used to test the hypotheses and which metrics were considered for each analysis. The strength (Mantel *r*) of decay was used to test hypothesis H_1_, and the rate of decay (slope) was used to hypotheses H_2_–H_4_

We gathered our data by directly contacting data owners or using the existing data sources, such as GrassPlot (Biurrun et al., 2021; Dengler et al., 2018), sPlot (Bruelheide et al., 2019) and CESTES (Jeliazkov et al., 2020). We included datasets that provided raw data of species abundances, functional traits, environmental variables and spatial coordinates of the study sites. A few datasets (*n* = 6) provided only species occurrence rather than abundance information (Supporting Information Appendix S1). The traits included in the datasets were chosen by data owners from a suite of traits that should respond well to environmental variation. For plant datasets compiled from the sPlot database, trait information was commonly derived from the TRY database (Kattge et al., 2011). Regarding the CESTES database, we compiled 48 datasets, specifically from: fish communities (Brind’Amour et al., 2011; Chong‐Seng et al., 2012; Cleary et al., 2016; Villéger et al., 2012), terrestrial vascular plants (Bagaria et al., 2012; Chmura et al., 2016; Eallonardo et al., 2013; Frenette‐Dussault et al., 2011; Fried et al., 2012; Jamil et al., 2013; van Klink et al., 2017; Meffert & Dziock, 2013; Pakeman, 2011; Raevel et al., 2012; Ribera et al., 2001; Robroek et al., 2017), aquatic macroinvertebrates (Díaz et al., 2007; Gallardo et al., 2009; Jeliazkov et al., 2014), terrestrial arthropods (Barbaro & van Halder, 2009; Barbosa et al., 2015; Bartonova et al., 2016; Dziock et al., 2011; Gibb et al., 2015; Gonҫalves‐Souza et al., 2014; van Klink et al., 2017; Krasnov et al., 2015; Lowe et al., 2018), birds (Barbaro et al., 2012, 2017; Barbaro & van Halder, 2009; Charbonnier et al., 2016; Cleary & Renema, 2007; Meffert & Dziock, 2013), bats (Charbonnier et al., 2016; Farneda et al., 2015), bryophytes (Robroek et al., 2017), butterflies (Bartonova et al., 2016; Robinson et al., 2014), corals (Rachello‐Dolmen & Cleary, 2007) and foraminifera (Cleary & Renema, 2007). We included only datasets with ≥10 sites, two environmental variables and three traits or trait categories. In some cases, more than one dataset, representing different taxonomic groups with different responses to the environment and dispersal abilities (e.g., stream macroinvertebrates and diatoms) was collected in the same study area. In total, 148 datasets representing 17 major biotic groups from terrestrial (*n* = 87), freshwater (*n* = 41) and marine (*n* = 21) environments were assembled, amounting to >17,000 study sites around the globe (Figure 2). Of the 148 datasets, 118 were published in peer‐reviewed journals (Appendix S1). Taxa were identified mostly to species or morphospecies; in a few cases, we used data at the genus level if existing taxonomic knowledge did not allow individual species to be distinguished. Within biotic groups, traits were generally the same or at least covered similar functional roles (Appendix S1). Finally, each dataset included (1) a sites‐by‐species abundance matrix; (2) a species‐by‐traits table; (3) a sites‐by‐spatial coordinates table; and (4) a sites‐by‐environmental variables table (Figure 3a). Detailed information about collected datasets can be found in Appendix S1 and information about data curation in Appendix S2. All the data curation and further analyses were performed in the software R v.4.0.2 using the appropriate R packages. We will consistently refer to the functions used and their respective packages from here on.

### 2.2 Community similarity

When estimating community similarities, we used both occurrence and abundance data, because occurrences are informative about the drivers and patterns of communities along geographical gradients, whereas abundances inform patterns along environmental gradients well (Declerck et al., 2011). The similarity in species composition between two communities (hereafter, taxonomic similarity) was estimated with the function *beta* in the package BAT v.2.7.0 (Cardoso et al., 2020). For the similarity in trait composition (hereafter, functional similarity), we first represented the ecological niche spaces of species as *n*‐dimensional hypervolumes (Hutchinson, 1957) *sensu* Blonder (2018). We used probabilistic hypervolumes based on the Gaussian kernel density estimator instead of other widely used methods (e.g., convex hulls; Villéger et al., 2013) because it allows the inclusion of abundance information in the estimation of functional space and it gives different probabilities to more or less populated areas of the functional space, being less influenced by outliers (Mammola & Cardoso, 2020; Mammola et al., 2021). Community similarity (*S*) ranges between zero and one and is commonly calculated for pairs of communities as the sum of the unique features of each community divided by the sum of the shared features between communities and the unique features of each community. We estimated the taxonomic and functional similarities between communities *j* and *k* using the Sørensen similarity index as:

(1) $$S_{\mathrm{jk}}=1‐\frac{b+c}{2a+b+c}$$

where *a* is the sum of the shared features between the communities *j* and *k*, *b* is the sum of the unique features of community *j*, and *c* is the unique features of community *k*. When estimating taxonomic similarities, each species is a feature that may or may not be shared by two communities (Figure 3b). For functional similarities, each feature comprises the area of the hypervolume that is either shared between two communities or unique to each community (Figure 3b).

To construct the hypervolume of each community, we first calculated the pairwise difference between species using the Gower similarity index. We used Gower similarity as modified by Bello et al. (2021) because it gives balanced weights for categorical, continuous, dummy and fuzzy‐coded variables. The modified gower distance was calculated with the function *gawdis* within the package gawdis v.0.1.3 (Bello et al., 2021). Then, we ran a principal coordinates analysis (PCoA) on this distance matrix and summarized the trait data in three PCoA axes, following a trade‐off between computation time and information quality. Using the three PCoA axes, we constructed the hypervolume using the function *kernel.build* from BAT. Finally, we estimated the amount of overlap between two hypervolumes, in addition to the unique area of each community, using the function *kernel.beta* of BAT that builds on *hypervolume_set* of hypervolume v.2.0.1 (Blonder & Harris, 2019). For functional similarities, if two communities do not share any species, taxonomic similarity would be, by definition, lower than functional similarity if any continuous trait (e.g., body size; Figure 3b) is included. Details of the calculation of similarities using the Sørensen‐based indices for occurrence and abundance data can be found in the (Appendix S2). The main results are given for occurrence data in the main text, whereas abundance‐based results can be found in the (Appendix S3).

### 2.3 Spatial and environmental distance

For each dataset, spatial distance was calculated as the geographical distance (in kilometres) between the pairs of sites using the function *earth.dist* of the package fossil v.0.4.0 (Vavrek, 2011) (Figure 3b). Environmental distance was calculated as the Euclidean distance between all the pairs of sites, considering the standardized environmental variables within each dataset (Figure 3b), with the function *vegdist* of the package vegan v.2.5–6 (Oksanen et al., 2019). From the original environmental variables in each dataset, we only kept the continuous variables with <5% missing values (for data curation, see Appendix S2). Given that the datasets contained different numbers and types of environmental variables, the values of environmental distance were context dependent and not very informative for comparison across datasets. We therefore assumed that the environmental gradient increased with spatial extent and rescaled the actual environmental distance to range between zero and one in each dataset by dividing actual values by the maximum environmental distance of the dataset.

### 2.4 Distance decay of similarity

To comply with the assumption of nonlinearity in the distance decays, the strength of the distance decay relationship was assessed by ranked Mantel tests (using a Spearman correlation, i.e., Mantel *r*). The rate of the decay was modelled as the slope of generalized linear models (GLMs) following a quasi‐binomial family with log‐link (Millar et al., 2011), representing a negative exponential curve between the community similarity and distance (Figure 3). One of the main assumptions of the distance decay is that the slope of the relationship should be negative (Nekola & McGill, 2014), and positive slopes suggest either periodicity in the environmental gradient or a mismatch between the communities and the measured environmental variables (Nekola & White, 1999). Therefore, whenever datasets showed positive distance decay slopes, these were transformed to zero values. In total, five datasets had positive slopes for taxonomic similarities, whereas 11 datasets had positive slopes for functional similarities.

### 2.5 Statistical analysis

We tested our master hypothesis using two different approaches. Firstly, we investigated whether taxonomic or functional distance decay is stronger along spatial and environmental distance (H_1_) by performing Student’s paired *t* tests to compare Mantel *r* drawn from taxonomic and functional similarity for each dataset, considering both spatial and environmental distance (Figure 3d). Secondly, we generated a null distribution of functional similarity values by randomizing the names of the species across the trait table 999 times. At each iteration, the functional similarities were calculated and regressed against spatial and environmental distance. The slopes of these relationships were used to obtain a null distribution of slopes under the assumption that the rate of functional decay is random given the rate of taxonomic decay. Deviations from null distribution were tested using standardized effect sizes (SES; Gotelli & Graves, 1996); SES values >1.96 indicate that functional similarity decays faster than expected given taxonomic decay, whereas SES values <−1.96 indicate that functional similarity decays slower than expected given taxonomic decay (Swenson et al., 2011).

We also investigated the ecological and geographical factors driving the rate of the distance decay across datasets. Each dataset was characterized with respect to: (1) latitude, recorded as the absolute mean value of all the sites of the dataset; (2) spatial extent, expressed as the largest pairwise distance (in kilometres) between study sites; (3) realm, classified into freshwater, marine and terrestrial environments; (4) body size information drawn from literature (Hillebrand, 2004; Peters, 1983), estimated as the mean log_10_‐transformed fresh weight (in grams) of the biotic group included in the dataset; (5) dispersal mode, classified as active and passive modes and organisms dispersed by seeds; (6) taxonomic γ‐diversity, expressed as the total number of species in the dataset; (7) functional γ‐diversity, measured as the total volume of the union of the *n*‐dimensional hypervolumes estimated within the dataset (Mammola & Cardoso, 2020); (8) total number of study sites in the dataset; and (9) the number of environmental variables in the dataset. For body sizes, we note that although the size range within the biotic group can be large (up to five orders of magnitude), it is small compared with the overall variation obtained across organism groups (12 orders of magnitude). For more details on body size approximations, see the papers by Hillebrand (2004) and Drakare et al. (2005). The taxonomic γ‐diversity was included to study whether there is a typical positive relationship between γ‐diversity (taxonomic and functional) and β‐diversity (Kraft et al., 2011; Lamanna et al., 2014). Functional γ‐diversity was estimated using the function *hypervolume_set* of the package hypervolume v.2.0.12 (Blonder & Harris, 2019). Hypervolumes are expressed in units of SDs to the power of the number of trait dimensions used (i.e., three). The number of study sites and the number of environmental variables for each dataset were included to explore their potential effect on distance decay.

Finally, we used boosted regression trees (BRTs) to test the effects of latitude (H_2_), spatial extent (H_3_) and realm (H_4_) on the rate of taxonomic and functional distance decay along spatial and environmental distance across the datasets. The BRT parameters were selected to amplify the deviance explained by the model. We tested interaction depths of 0.1, 0.01 and 0.001, and learning rates between two and five. The best models were the ones with a learning rate of five and interaction depth of 0.001. Given that the datasets in this study have not always followed the same sampling methodology and show different functional traits and environmental variables, we fitted the BRT models following a Laplace distribution of the errors to reduce the absolute error loss from the variation among datasets. We included taxonomic and functional γ‐diversity, number of sites and number of environmental variables in the dataset as predictor variables for controlling the heterogeneity across datasets within the model (Figure 3d). The BRT models were fitted using the function *gbm.step* of the package dismo v.1.1–4 (Hijmans et al., 2017). Given that some predictors could show cross‐information (e.g., marine ecosystems have smaller organisms than terrestrial systems in our datasets), we tested whether there were significant interactions between predictors in BRTs using the functions *gbm.interaction*. For understanding how *gbm.interaction* works and for more in‐depth details about BRTs, we refer to the (Appendix S2 and references therein). Additionally, we performed sensitivity analysis to ensure that the patterns detected were not an artefact of sample size. In the sensitivity analysis, models were refitted using subsampled data with 90, 70 and 50% of the full observations.

We partitioned the similarities into replacement and richness difference components following the methodology described in the (Appendix S2). Replacement gives the variation resulting from the substitution of species (taxonomic replacement) or functional traits (functional replacement), whereas richness differences account for the variation resulting from net differences induced by the loss/gain of species or traits (Carvalho et al., 2012). We showed the full results of the distance decay using occurrence‐based total similarities (Equation 1), but we also used abundance‐based similarities and showed their main findings in the main text, with further details in the (Appendix S3). To keep the narrative concise, in the main text we show only the results of the partitioned components using occurrence data. All figures were built using the packages from the tidyverse suite (Wickham et al., 2019).

## 3 RESULTS

### 3.1 Strength of the distance decay

**FIGURE 4:**
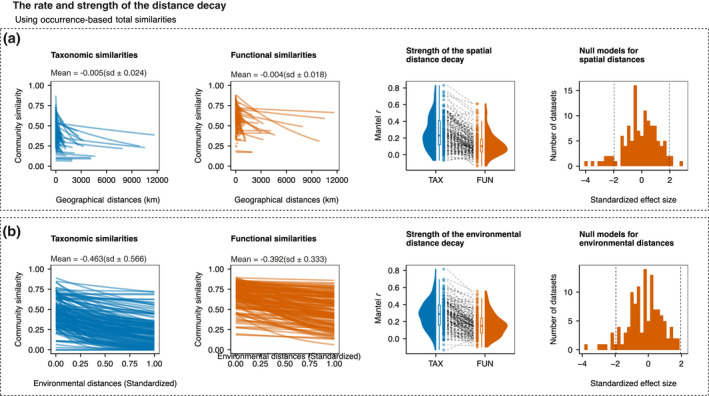
The distance decay along (a) spatial distance and (b) environmental distance. The light blue lines show the distance decay of taxonomic similarity, and the orange lines show the distance decay of functional similarity. The first and second columns show the rate (slope) of the taxonomic and functional distance decay, respectively; the third column shows the strength (Mantel *r*) of the distance decay of taxonomic and functional similarities; and the fourth column shows the standardized effect sizes of the slopes of each dataset

The distance decays showed a wide range of shapes, from very steep decays to almost flat relationships (Figure 4). The average Mantel *r* using occurrence data along spatial distance for taxonomic similarities was .254 (SD ±0.197) and .115 (SD ±0.143) for functional similarities. Spatial distance decays of taxonomic similarities were significantly stronger than the distance decays of functional similarities when considering both occurrence (Figure 4a; *t* = 13.124, *p* < .001, d.f. = 146) and abundance data (Appendix S3; Figure S3.3), supporting H_1a_ that spatial distance decay is stronger for taxonomic than functional similarities (Figure 4a). In 31 datasets, the spatial distance decay of functional similarities was stronger than taxonomic similarities.

However, our results did not support H_1b_, because the distance decay for taxonomic similarities (mean Mantel *r* = .272, SD ±0.176) was also, on average, stronger than for functional similarities (mean Mantel *r* = .150, SD ±0.144) along environmental distance, considering both occurrence (Figure 4b; *t* = 10.342, *p* < .001, d.f. = 146) and abundance data (Appendix S3; Figure S3.3). Note, however, that 32 of 148 datasets had stronger distance decay of functional similarities than taxonomic similarities along environmental gradients.

### 3.2 Rate of the distance decay

The mean slope of the spatial distance decay was −0.009 (SD ±0.028) for taxonomic similarities and −0.004 (SD ±0.020) for functional similarities (Figure 4a). Null models showed that only 13 datasets had a functional distance decay significantly different from expected given taxonomic decay, with three datasets having faster slopes and 10 having slower slopes (Figure 4a). For environmental distance, the mean slope of the distance decay was −1.077 (SD ±1.066) for taxonomic similarities and −0.355 (SD ±0.031) for functional similarities (Figure 4b). Null models along environmental distance showed that only 11 datasets had a functional distance decay significantly different from expected given the taxonomic decay, from which only one was slower (Figure 4b).

**FIGURE 5:**
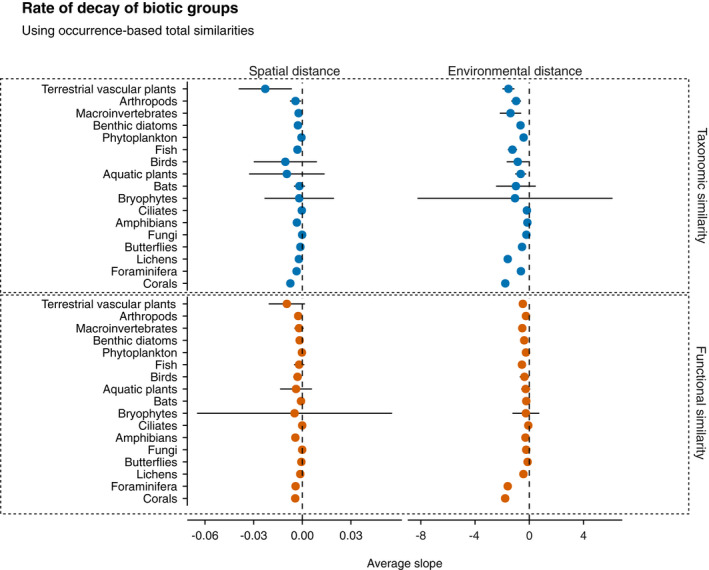
The average rate of decay (slopes) of biotic groups using occurrence data along spatial and environmental distance. The vertical dashed lines highlight the zero rate (absence of decay), and the horizontal lines indicate the standard deviation of the mean. The blue circles show the rate of decay of taxonomic similarities, and the orange circles show the rate of decay of functional similarities. Large error bars are attributable to low sample size (i.e., a low number of datasets for a given taxon)

Regarding the biotic groups, terrestrial plants had the steepest slopes along spatial distance for both taxonomic and functional similarities (Figure 5). Along environmental distance, corals had the steepest slopes along spatial distance, whereas foraminifera had the steepest slopes along environmental distance (Figure 5). Similar patterns were found for abundance‐based similarities, except for the biotic groups, where aquatic plants had the steepest slopes along spatial distance (Appendix S3; Figure S3.4).

**FIGURE 6:**
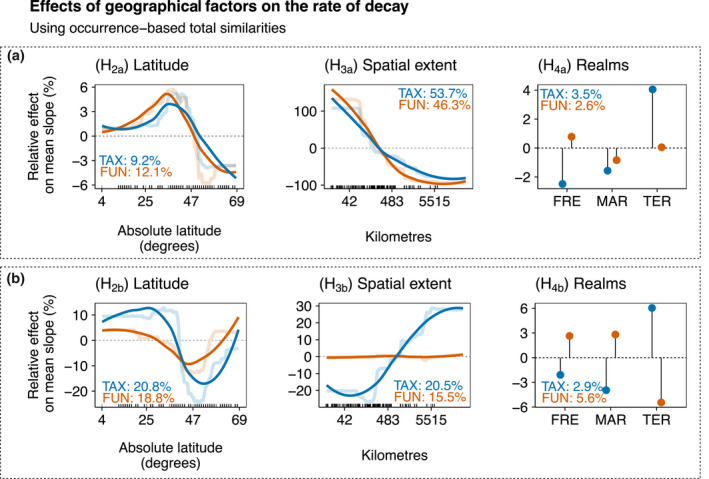
Relative effects (expressed as percentages) of geographical factors on the rate of decay along (a) spatial distance decay and (b) environmental distance decay of the total component of taxonomic (TAX, light blue) and functional (FUN, orange) similarities using occurrence data across datasets. Partial dependence plots show the effects of a predictor variable on the response variable after accounting for the average effects of all other variables in the model. Positive values indicate an increase in the rate of decay (steeper slopes) compared with the mean rate, whereas negative values indicate a decrease in the rate of decay (flatter slopes) compared with the mean rate. Semi‐transparent lines represent the actual predicted effects; continuous lines represent LOESS fits to predicted values from boosted regression trees (BRTs). We show here only the variables related to the specific hypotheses [i.e., latitude, spatial extent and realms (FRE = freshwater; MAR = marine; TER = terrestrial)]

**FIGURE 7:**
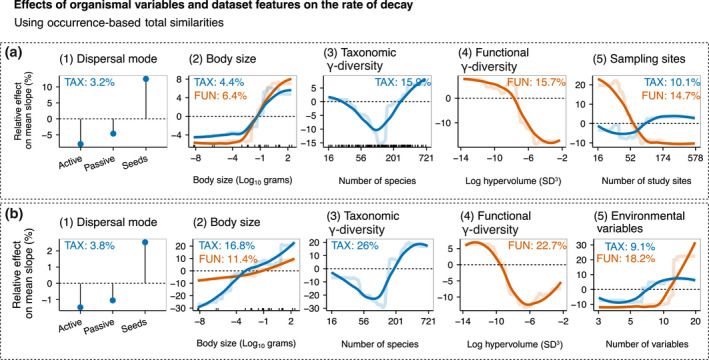
Relative effects (expressed as a percentage) of organismal variables and dataset features on the rate of decay along (a) spatial distance and (b) environmental distance, considering the total component of taxonomic (light blue lines) and functional (orange lines) similarities using occurrence data across datasets. Partial dependence plots show the effects of a predictor variable on the response variable after accounting for the average effects of all other variables in the model. Positive values indicate an increase in the rate of decay (steeper slopes) compared with the mean rate, whereas negative values indicate a decrease in the rate of decay (flatter slopes) compared with the mean rate. Semi‐transparent lines represent the actual predicted effects; continuous lines represent LOESS fits to predicted values from boosted regression trees (BRTs). We show here the organismal variables and the variables related to the dataset features

Across datasets, BRT explained 35.4% of the deviance of the slopes of the spatial distance decay for taxonomic similarities and 38.3% for functional similarities using occurrence data. For the distance decay along environmental distance, BRT explained only 14.4% of the deviance of the slopes of the decay of taxonomic similarities and 6.6% for functional similarities. Spatial extent, latitude and γ‐diversity contributed the most to the variation in slopes along either spatial or environmental distance using both occurrence‐ and abundance‐based similarities (Figures 6a and 7a; Appendix S3; Figures S3.5 and S3.6). We found interactions for the BRT only on taxonomic similarities along environmental gradients. These were between: (1) taxonomic γ‐diversity and body size (interaction size = 5.09); and (2) spatial extent and latitude (interaction size = 3.79). These are probably related to the higher number of species included in the datasets containing small biotic groups and the fact that larger datasets had a mean latitude near the equator. There was no evident effect of sample size on the main patterns according to the sensitivity analysis (Appendix S2; Figures S2.1 and S2.2).

### 3.3 Latitudinal patterns

The slopes of spatial distance decay of both taxonomic and functional similarities were steepest in datasets centred at *c*. 35–45°, providing only partial support for H_2a_ that distance decay was flatter at high latitudes (Figure 6a). Note that spatial distance decay decreased sharply towards the poles. The slopes of environmental distance decay were flattest in the datasets at *c*. 50° (Figure 6b). However, note that functional distance decay increased towards the poles, providing partial support to hypothesis H_2b_. Similar patterns were found for abundance‐based similarities (Appendix S3; Figure S3.5).

### 3.4 Spatial extent

The distance decay of taxonomic and functional similarities was flatter in the datasets that covered a larger spatial extent for both occurrence (Figure 6a) and abundance data (Appendix S3; Figure S3.5a), supporting hypothesis H_3a_ that distance decay becomes flatter with increasing spatial extent. For environmental distance, distance decay was steeper in the datasets that covered larger spatial extents only for taxonomic similarities, but functional distance decay did not vary with extent. Thus, our results agreed, in part, with H_3b_ that distance decay would become steeper with larger spatial extent.

### 3.5 Realms

Marine ecosystems typically had flatter slopes in comparison to freshwater or terrestrial ecosystems, thus agreeing with H_4_ (Figure 6). However, the importance of the realms in BRTs was low overall. A similar pattern emerged for abundance‐based similarities (Appendix S3; Figure S3.5).

### 3.6 Organismal variables and dataset features

Organisms relying on seed dispersal had steeper slopes along spatial and environmental distance than other dispersal types, but the overall importance of dispersal mode was low (Figure 7b). The slopes of both spatial and environmental distance decays were steeper for larger‐bodied organisms in taxonomic and functional similarities (Figure 7a,b). Taxonomic γ‐diversity had a U‐shaped relationship with slopes for distance decay along spatial and environmental distance (Figure 7b). Slopes of distance decay had an overall flattening trend towards higher functional γ‐diversity for both spatial and environmental distance (Figure 7a,b). Generally, taxonomic slopes were steeper in the datasets where the number of study sites was higher (Figure 7a), whereas the opposite was true for functional slopes. The slopes were flatter when datasets contained only a few environmental variables (Figure 7b).

### 3.7 Replacement and richness differences

The slopes of taxonomic and functional replacement along spatial distance decreased rapidly in the datasets above 35° (Appendix S4; Figure S4.7a). Along environmental distance, the taxonomic replacement increased towards higher latitudes, whereas the functional replacement had a U‐shaped pattern, with a decrease from low to mid‐latitudes (*c*. 45°) and a sharp increase towards the poles (Appendix S4; Figure S4.7b). For the richness differences component, the slopes of taxonomic similarities were steepest in the datasets at *c*. 45° for the spatial distance decay, whereas the slopes of functional similarities became notably steeper with latitude (Appendix S4; Figure S4.8a). For environmental distance, slopes became flatter from low to high latitudes up to *c*. 50° for taxonomic similarities, whereas for functional similarities, slopes did not vary along latitude (Appendix S4; Figure S4.8b). Both replacement and richness differences showed flatter spatial slopes with increasing spatial extent (Appendix S4; Figure S4.7 and S4.8). In contrast, environmental slopes became steeper with spatial extent for taxonomic replacement and flattened for functional replacement (Appendix S4; Figure S4.7b), whereas the functional slopes showed an opposite pattern (Appendix S4; Figure S4.8b). Furthermore, marine ecosystems showed the flattest slopes for taxonomic replacement along environmental gradients (Appendix S4; Figure S4.7b), whereas terrestrial ecosystems had the flattest slopes for richness differences (Appendix S4; Figure S4.8b). Details about the organismal variables and dataset features can be found in the (Appendix S4; Figures S4.9 and S4.10).

## 4 DISCUSSION

Community ecology, macroecology and biogeography have lacked a comprehensive evaluation of functional β‐diversity across different taxa and ecosystems globally. Earlier studies suggest that functional β‐diversity better reflects environmental variability compared with taxonomic β‐diversity and that focusing on functional β‐diversity might help us, for example, to gain an understanding of how humans impact ecosystems by modifying the local environment (Meynard et al., 2011; Sokol et al., 2011; Spasojevic et al., 2014; Weinstein et al., 2014). This is because functional traits should reflect best the ecological requirements of species. Using a comparative analysis across biotic groups, ecosystem types and realms, we show here that taxonomic distance decay is generally stronger along spatial gradients than functional distance decay and that the decay of functional similarities along environmental gradients is typically not stronger than the decay of taxonomic similarities, unlike previous suggestions.

### 4.1 The strength of the distance decay of taxonomic and functional similarities

The stronger signal of taxonomic than functional distance decay along space provides empirical evidence that taxonomic distance decay is a robust approach for ecological and biogeographical studies, supporting H_1a_. Compositional differences effectively summarize dispersal‐related factors in addition to species responses to climatic and other spatially structured environmental variables. However, spatial distance decay of functional similarities might not reflect the geographical differences in biotic communities well. This is likely to stem from the different roles played by deterministic and stochastic factors shaping community composition, because it has been shown that dispersal limitation or species pool effects should be more important for taxonomic than for functional composition (Soininen et al., 2016). Some morphological or morphometric traits are informative when exploring geographical patterns in functional composition (Soininen et al., 2016); for example, seed mass and wood density explained the variability of tree communities along broad spatial gradients better than species identity alone (Siefert et al., 2013). The type of dispersal is also an important trait to include when assessing community‐level patterns along spatial gradients (Bie et al., 2012). Unsurprisingly, we found that the datasets with larger spatial extent and species pool size were more likely to have a stronger distance decay of taxonomic than functional similarities (Appendix S5; Table S5.1). We argue that when the study extent is large it might cross several species pools, and it is more likely that species occurrences are also affected by historical and dispersal‐related factors and not only by environmental preferences (Soininen et al., 2016). Furthermore, we found that passive dispersers and datasets with higher functional γ‐diversity were less likely to have stronger decay of taxonomic similarities than functional similarities. In our datasets, passive dispersers were microorganisms and, therefore, efficient dispersers with a good ability to reaching sites with suitable environmental conditions (Bie et al., 2012; Fontaneto, 2019). Thus, for these taxa, functional distance decay should be more informative than taxonomic distance decay.

Regarding environmental gradients, functional distance decay was also weaker than taxonomic distance decay. Although counterintuitive, this relationship has been found previously (Heino & Tolonen, 2017; da Silva & Hernández, 2015; Teittinen & Virta, 2021). For example, Teittinen and Virta (2021) observed stronger distance decay of taxonomic than functional similarities along environmental gradients, which they attributed to the greater number of species than functional traits in their data. Also, Heino and Tolonen (2017) found similar results for macroinvertebrate communities of boreal lakes and related it to the trait resolution, which could probably be improved by the addition of several other physiological traits relevant for the organisms in question. Here, additional analysis showed that increasing spatial extent, species pool and the number of environmental variables significantly increased the probability of a dataset having stronger decay of taxonomic similarities compared with functional similarities (Appendix S5; Table S5.2). In fact, the ratio between taxonomic and functional decay depends on whether the species replaced from one community to another are a random subsample of functionally redundant species from the regional pool (Swenson et al., 2011). Also, species pool size and functional redundancy typically exhibit a positive correlation (Cannicci et al., 2021; Mouillot et al., 2014), which, in turn, should increase the functional similarities between sites (Jarzyna & Jetz, 2018). We suggest that within a large species pool, the functional redundancy of species increases, given the limited set of trait combinations and/or available niches. Therefore, smaller species pools are more likely to have functionally unique species and lower functional similarities than larger pools. In the case of large pools, we found that taxonomic decay was often stronger than functional decay. Furthermore, because species pool size increases with study extent (Drakare et al., 2005; Palmer & White, 1994; Triantis et al., 2011), the datasets with larger extents had slower functional distance decay even along environmental gradients, and taxonomic composition turned out to be the best descriptor of distance decay patterns. Another possible reasoning is that filtering on a given trait might filter other traits concomitantly, and if the focal trait is not included in the analyses, a mismatch between functional composition and the environment is expected. On the contrary, a dataset might comprise traits not affected by the environment, which tends to increase the functional similarity among sites. Therefore, because functional diversity patterns depend strongly on the traits measured (Zhu et al., 2017), the choice of traits should be planned carefully.

### 4.2 The effect of latitude on the rate of distance decay

In addition to our master hypothesis, we investigated whether the rate of distance decay showed consistent variation across realms, along geographical gradients and among major taxonomic groups. We did not find slower rates of decay along spatial distances in the datasets at higher latitudes, but we found a unimodal relationship with the highest decay rate at *c*. 30°. Similar results have been found earlier in terrestrial vertebrates when considering only the turnover component of β‐diversity (Castro‐Insua et al., 2016) and for the total β‐diversity of marine phytoplankton (Martin et al., 2021). It is noteworthy that our latitudinal patterns were related mainly to the replacement component for both taxonomic and functional decay (Appendix S4; Figure S4.7). Regarding environmental gradients, we found opposing patterns compared with spatial gradients, with the flattest rates of decay in the datasets near 50° and a notable increase from 60° towards the poles. A hump‐shaped relationship between functional diversity and latitude has also been found previously for aquatic macroinvertebrates (Múrria et al., 2020), also with the minimum at *c*. 50°. Múrria et al. (2020) were studying patterns in functional dispersion, whereas we found here that the breakpoint was related mainly to the differences in richness for taxonomic similarities and replacement for functional similarities.

Traditionally, latitudinal patterns of biodiversity have been explained by Rapoport's rule, positing that there is an increase in species range size towards high latitudes (Stevens, 1989), hence lower taxonomic replacement. However, the breakpoints found in our data suggest that some additional factors might have generated the patterns. For example, landscape fragmentation might increase β‐diversity (Jamoneau et al., 2012), especially at mid‐latitudes that showed the highest levels of human impact in this study (Halpern et al., 2008; Venter et al., 2016). Also, it has been suggested previously that the distance decay along spatial distances is stronger at mid‐latitudes than at the poles because northern communities result from postglacial recolonization processes, flattening distance decay relationships (Gómez‐Rodríguez & Baselga, 2018). Although inferring processes from observational data is difficult (Cadotte & Tucker, 2017), we would like to speculate on some possible mechanisms generating our breakpoint patterns. Strong seasonality, resource scarcity and climatic stress should select only the highly specialized taxa and modify the functional space towards the poles (Lamanna et al., 2014). Therefore, it is plausible that the climatic stress leads to an increase in richness differences in communities towards the poles, as observed in vertebrates elsewhere (Castro‐Insua et al., 2016). Moreover, as environmental heterogeneity increases towards the poles, and functional clustering is expected to be stronger at higher latitudes (Jarzyna et al., 2021; but see Kruk et al., 2017), we suggest that strong environmental filtering in datasets at higher latitudes (above 50°) selects for the species with different trait combinations between sites, thereby increasing the rate of functional decay. The latitudinal decrease in the rate of abundance‐based functional distance decay (Appendix S3; Figure S3.3) is further evidence of an optimal utilization of the functional space, as has been observed earlier exclusively for marine organisms (Edie et al., 2018). However, these potential explanations should be tested further.

### 4.3 The effect of spatial extent on the rate of distance decay

The rate of spatial distance decay was slower in the datasets covering a larger spatial extent, suggesting that regional species pools are limited and that new species are not found constantly at the same frequency when extent is larger. Lower decay rates in larger study areas could also result from repeated patterns in environmental variation; that is, environmental patchiness or natural periodicity in the environment (Nekola & White, 1999). In agreement with our hypothesis, we also found that the rate of taxonomic decay along environmental distance was higher in the datasets covering a larger spatial extent. These findings indicate that spatial distance decay is more affected by species pool effects and dispersal processes than environmental distance decay, possibly because the latter reflects more strongly the level of local deterministic environmental filtering processes. Similar evidence has accumulated from case studies conducted in various ecosystems (Meynard et al., 2011; Sokol et al., 2011; Weinstein et al., 2014; Zagmajster et al., 2014). The finding that the rate of distance decay along environmental distance was higher in the datasets covering larger extents indicates the stronger environmental filtering for larger study areas. We also note that, in our BRT models, the extent, latitude and γ‐diversity had by far the largest relative importance, suggesting that their interplay shapes distance decay to a great extent.

### 4.4 The effect of realm on the distance decay

We found evidence for a lower rate of distance decay in marine versus terrestrial or freshwater ecosystems. Overall, this finding agrees with an earlier meta‐review on β‐diversity (Soininen, McDonald, et al., 2007), suggesting that large‐scale diversity patterns are generally weaker in marine ecosystems (Bierne et al., 2003). Given that connectivity, energy flows, dispersal modes, body size structure and trophic dynamics differ substantially between dry and wet ecosystems (Shurin et al., 2006), it is vital to investigate possible differences in turnover among the realms more closely.

### 4.5 Organismal variables and dataset features

Organism size did seem to affect taxonomic or functional distance decay along spatial and environmental gradients, because the slopes typically increased with organism body size. This might be because β‐diversity should be low among the small microbial taxa with efficient passive dispersal (Soininen, McDonald, et al., 2007). The rationale behind this idea is that efficient dispersal homogenizes communities among sites, resulting in lower β‐diversity (Mouquet & Loreau, 2003). Body size is also a key driver of the biological complexity of organisms (Heim et al., 2017), and it might be that smaller organisms show a much more limited set of trait combinations than macroorganisms, leading to a lower functional redundancy among larger species. Furthermore, our knowledge about the taxonomy and functional traits of organisms is typically size dependent. For example, the identification of larger species is much easier than that of microorganisms, which also applies to the identification and measurement of soft functional traits (Hodgson et al., 1999; Martínez et al., 2021). Therefore, the values of functional β‐diversity of small organisms might typically be underestimated.

Patterns in environmental distance decay were relatively congruent with spatial distance decay regarding dispersal mode, suggesting that taxa dispersing passively do not seem to track environmental gradients more efficiently compared with less dispersive taxa. It might also be that small‐sized taxa were filtered along some unmeasured spatially structured environmental gradients, and the pattern was thus detected as spatial turnover even if caused by some underlying unmeasured environmental factors. Forthcoming studies would benefit greatly from disentangling the signal of unmeasured environmental variables from true dispersal limitation (Stegen et al., 2013).

### 4.6 Study design

There are also some possibly influential aspects in our study design that should be discussed. Although the study is global in its extent, the availability of datasets was not evenly distributed geographically. This is a well‐known problem in biodiversity research (Titley et al., 2017) that calls for complementary studies to verify that these trends hold true in poorly sampled regions. Also, we relied on the suite of traits and environmental variables included in the original datasets, hence the collection of traits and environmental variables used differed somewhat among datasets even for the same focal taxonomic groups. Although traits covered mostly the same functional roles of the species, the variation in traits and environmental variables across datasets increases the uncertainty on how environmental variables filter the functional structure of communities in different contexts and how strong the community–environment relationships might be. An alignment of key traits and environmental variables is therefore desirable but requires a suite of sister studies following the same protocol, which is, unfortunately, not yet available. Moreover, the fact that some of the biotic groups (e.g., bryophytes, corals, foraminifera) were underrepresented in our analysis, with only a few datasets included (Figure 2), or the total lack of some taxa (e.g., bacteria, and aquatic and terrestrial mammals), makes it more difficult to generalize distance decay across certain taxa.

### 4.7 Concluding remarks

We believe our analysis is an important step towards a more comprehensive understanding of patterns and drivers of functional β‐diversity, particularly in comparison to the patterns and drivers of taxonomic β‐diversity that have so far attracted much more research interest. Here, we found that functional distance decay is scale dependent and a product of large‐scale geographical factors (latitude) and taxonomic and functional γ‐diversity but is also driven by the biology of organisms to some degree. In general, taxonomic distance decay is a useful tool for many aspects of biogeographical research because it reflects dispersal‐related factors in addition to species responses to climatic and other spatially structured environmental variables. However, functional distance decay might be a cost‐effective option for investigating how species respond to the environment, especially for microorganisms (e.g., microalgae), which are typically difficult to identify to the species level. Overall, the present findings and data shed light into the congruence between the functional and taxonomic diversity patterns globally and provide useful new information to the field of functional biogeography.

## CONFLICT OF INTEREST

The authors declare no conflicts of interest.

## AUTHOR CONTRIBUTIONS

Caio Graco‐Roza and Janne Soininen contributed equally to the original idea, data analysis and the writing of the first draft. Jani Heino and Otso Ovaskainen advised on the main idea, analysis and commented on the first draft. Francesco Maria Sabatini coordinated the data compilation from the “sPlot” database and commented on the first draft. Martin Gossner coordinated the compilation of the data from the “Biodiversity Exploratories” project and commented on the first draft. All other authors shown in alphabetical order contributed data and commented on the draft.

## Supporting information

Appendix S1

Appendix S2

Appendix S3

Appendix S4

Appendix S5

## Data Availability

All the data used in this study can be found in a Zenodo repository (https://doi.org/10.5281/zenodo.6406911). Code to run the analysis is available in a GitHub repository (https://github.com/graco‐roza/DISTANCE_DECAY_2.0). The authors declare that some datasets were compiled from sPlot (https://www.idiv.de/en/splot.html), Biodiversity Exploratories (https://www.biodiversity‐exploratories.de/en/) and CESTES database (Jeliazkov et al., 2020).

## References

[geb13513-bib-0001] Anderson, M. J. , Crist, T. O. , Chase, J. M. , Vellend, M. , Inouye, B. D. , Freestone, A. L. , Sanders, N. J. , Cornell, H. V. , Comita, L. S. , Davies, K. F. , Harrison, S. P. , Kraft, N. J. B. , Stegen, J. C. , & Swenson, N. G. (2011). Navigating the multiple meanings of β diversity: A roadmap for the practicing ecologist. Ecology Letters, 14, 19–28. 10.1111/j.1461-0248.2010.01552.x 21070562

[geb13513-bib-0002] Astorga, A. , Oksanen, J. , Luoto, M. , Soininen, J. , Virtanen, R. , & Muotka, T. (2012). Distance decay of similarity in freshwater communities: Do macro‐ and microorganisms follow the same rules? Global Ecology and Biogeography, 21, 365–375. 10.1111/j.1466-8238.2011.00681.x

[geb13513-bib-0003] Bagaria, G. , Pino, J. , Rodà, F. , & Guardiola, M. (2012). Species traits weakly involved in plant responses to landscape properties in Mediterranean grasslands. Journal of Vegetation Science, 23, 432–442. 10.1111/j.1654-1103.2011.01363.x

[geb13513-bib-0004] Barbaro, L. , Brockerhoff, E. G. , Giffard, B. , & van Halder, I. (2012). Edge and area effects on avian assemblages and insectivory in fragmented native forests. Landscape Ecology, 27, 1451–1463. 10.1007/s10980-012-9800-x

[geb13513-bib-0005] Barbaro, L. , Rusch, A. , Muiruri, E. W. , Gravellier, B. , Thiery, D. , & Castagneyrol, B. (2017). Avian pest control in vineyards is driven by interactions between bird functional diversity and landscape heterogeneity. Journal of Applied Ecology, 54, 500–508. 10.1111/1365-2664.12740

[geb13513-bib-0006] Barbaro, L. , & van Halder, I. (2009). Linking bird, carabid beetle and butterfly life‐history traits to habitat fragmentation in mosaic landscapes. Ecography, 32, 321–333. 10.1111/j.1600-0587.2008.05546.x

[geb13513-bib-0007] Barbosa, E. P. , Lopes, S. M. , Kaminski, L. A. , Shimizu, G. H. , Gonc, T. , Santos, A. J. , Romero, G. Q. , Ecologia, D. , Paulista, U. E. , Nacional, M. , de Blattaria, S. , de Janeiro, R. , Mar, P. , Horizonte, B. , Gonçalves‐Souza, T. , Araújo, M. S. , Barbosa, E. P. , Lopes, S. M. , Kaminski, L. A. , … Romero, G. Q. (2015). Fine‐scale beta‐diversity patterns across multiple arthropod taxa over a neotropical latitudinal gradient. Biotropica, 47, 588–594. 10.1111/btp.12242

[geb13513-bib-0008] Bartonova, A. , Benes, J. , Fric, Z. F. , Chobot, K. , & Konvicka, M. (2016). How universal are reserve design rules? A test using butterflies and their life history traits. Ecography, 39, 456–464. 10.1111/ecog.01642

[geb13513-bib-0009] Bie, T. , Meester, L. , Brendonck, L. , Martens, K. , Goddeeris, B. , Ercken, D. , Hampel, H. , Denys, L. , Vanhecke, L. , Gucht, K. , Wichelen, J. , Vyverman, W. , Declerck, S. A. J. J. , de Bie, T. , de Meester, L. , Brendonck, L. , Martens, K. , Goddeeris, B. , Ercken, D. , … Declerck, S. A. J. J. (2012). Body size and dispersal mode as key traits determining metacommunity structure of aquatic organisms. Ecology Letters, 15, 740–747. 10.1111/j.1461-0248.2012.01794.x 22583795

[geb13513-bib-0010] Bierne, N. , Bonhomme, F. , & David, P. (2003). Habitat preference and the marine‐speciation paradox. Proceedings of the Royal Society of London. Series B: Biological Sciences, 270, 1399–1406. 10.1098/rspb.2003.2404 PMC1691380 12965032

[geb13513-bib-0011] Biurrun, I. , Pielech, R. , Dembicz, I. , Gillet, F. , Kozub, Ł. , Marcenò, C. , Reitalu, T. , Van Meerbeek, K. , Guarino, R. , Chytrý, M. , Pakeman, R. J. , Preislerová, Z. , Axmanová, I. , Burrascano, S. , Bartha, S. , Boch, S. , Bruun, H. H. , Conradi, T. , De Frenne, P. , … Dengler, J. (2021). Benchmarking plant diversity of Palaearctic grasslands and other open habitats. Journal of Vegetation Science, 32. 10.1111/jvs.13050

[geb13513-bib-0012] Blonder, B. (2018). Hypervolume concepts in niche‐ and trait‐based ecology. Ecography, 41, 1441–1455. 10.1111/ecog.03187

[geb13513-bib-0013] Blonder, B. , & Harris, D. J. (2019). hypervolume: High dimensional geometry and set operations using kernel density estimation, support vector machines, and convex hulls. https://cran.r‐project.org/package=hypervolume

[geb13513-bib-0014] Blowes, S. A. , Supp, S. R. , Antão, L. H. , Bates, A. , Bruelheide, H. , Chase, J. M. , Moyes, F. , Magurran, A. , McGill, B. , Myers‐Smith, I. H. , Winter, M. , Bjorkman, A. D. , Bowler, D. E. , Byrnes, J. E. K. K. , Gonzalez, A. , Hines, J. , Isbell, F. , Jones, H. P. , Navarro, L. M. , … Dornelas, M. (2019). The geography of biodiversity change in marine and terrestrial assemblages. Science, 366, 339–345. 10.1126/science.aaw1620 31624208

[geb13513-bib-0015] Brind’Amour, A. , Boisclair, D. , Dray, S. , Legendre, P. , Daniel, B. , Dray, S. , & Legendre, P. (2011). Relationships between species feeding traits and environmental conditions in fish communities: A three‐matrix approach. Ecological Applications, 21, 363–377. 10.1890/09-2178.1 21563569

[geb13513-bib-0016] Bruelheide, H. , Dengler, J. , Jiménez‐Alfaro, B. , Purschke, O. , Hennekens, S. M. , Chytrý, M. , Pillar, V. D. , Jansen, F. , Kattge, J. , Sandel, B. , Aubin, I. , Biurrun, I. , Field, R. , Haider, S. , Jandt, U. , Lenoir, J. , Peet, R. K. , Peyre, G. , Sabatini, F. M. , … Zverev, A. (2019). sPlot – A new tool for global vegetation analyses. Journal of Vegetation Science, 30, 161–186. 10.1111/jvs.12710

[geb13513-bib-0017] Buisson, L. , Grenouillet, G. , Villéger, S. , Canal, J. , & Laffaille, P. (2013). Toward a loss of functional diversity in stream fish assemblages under climate change. Global Change Biology, 19, 387–400. 10.1111/gcb.12056 23504778

[geb13513-bib-0018] Cadotte, M. W. , & Tucker, C. M. (2017). Should environmental filtering be abandoned? Trends in Ecology & Evolution, 32, 429–437. 10.1016/j.tree.2017.03.004 28363350

[geb13513-bib-0019] Cannicci, S. , Lee, S. Y. , Bravo, H. , Cantera‐Kintz, J. R. , Dahdouh‐Guebas, F. , Fratini, S. , Fusi, M. , Jimenez, P. J. , Nordhaus, I. , Porri, F. , & Diele, K. (2021). A functional analysis reveals extremely low redundancy in global mangrove invertebrate fauna. Proceedings of the National Academy of Sciences of United States of America, 118(32). 10.1073/pnas.2016913118 PMC8364210 34312251

[geb13513-bib-0020] Cardinale, B. J. , Duffy, J. E. , Gonzalez, A. , Hooper, D. U. , Perrings, C. , Venail, P. , Narwani, A. , Mace, G. M. , Tilman, D. , Wardle, D. A. , Kinzig, A. P. , Daily, G. C. , Loreau, M. , Grace, J. B. , Larigauderie, A. , Srivastava, D. S. , & Naeem, S. (2012). Biodiversity loss and its impact on humanity. Nature, 486, 59–67. 10.1038/nature11148 22678280

[geb13513-bib-0021] Cardoso, P. , Mammola, S. , Rigal, F. , & Carvalho, J. C. (2020). BAT: biodiversity assessment tools. https://CRAN.R‐project.org/package=BAT

[geb13513-bib-0022] Carvalho, J. C. , Cardoso, P. , & Gomes, P. (2012). Determining the relative roles of species replacement and species richness differences in generating beta‐diversity patterns. Global Ecology and Biogeography, 21, 760–771. 10.1111/j.1466-8238.2011.00694.x

[geb13513-bib-0023] Carvalho, J. C. , Malumbres‐Olarte, J. , Arnedo, M. A. , Crespo, L. C. , Domenech, M. , & Cardoso, P. (2020). Taxonomic divergence and functional convergence in Iberian spider forest communities: Insights from beta diversity partitioning. Journal of Biogeography, 47, 288–300. 10.1111/jbi.13722

[geb13513-bib-0024] Castro‐Insua, A. , Gómez‐Rodríguez, C. , & Baselga, A. (2016). Break the pattern: Breakpoints in beta diversity of vertebrates are general across clades and suggest common historical causes. Global Ecology and Biogeography, 25, 1279–1283. 10.1111/geb.12507

[geb13513-bib-0025] Charbonnier, Y. M. , Barbaro, L. , Barnagaud, J.‐Y.‐Y. , Ampoorter, E. , Nezan, J. , Verheyen, K. , & Jactel, H. (2016). Bat and bird diversity along independent gradients of latitude and tree composition in European forests. Oecologia, 182, 529–537. 10.1007/s00442-016-3671-9 27312262

[geb13513-bib-0026] Chmura, D. , Żarnowiec, J. , & Staniaszek‐Kik, M. (2016). Interactions between plant traits and environmental factors within and among montane forest belts: A study of vascular species colonising decaying logs. Forest Ecology and Management, 379, 216–225. 10.1016/j.foreco.2016.08.024

[geb13513-bib-0027] Chong‐Seng, K. M. , Mannering, T. D. , Pratchett, M. S. , Bellwood, D. R. , & Graham, N. A. J. J. (2012). The influence of coral reef benthic condition on associated fish assemblages. PLoS One, 7, 1–10. 10.1371/journal.pone.0042167 PMC3411644 22870294

[geb13513-bib-0028] Clarke, A. (1992). Is there a latitudinal diversity cline in the sea? Trends in Ecology & Evolution, 7, 286–287. 10.1016/0169-5347(92)90222-W 21236034

[geb13513-bib-0029] Cleary, D. , Polónia, A. , Renema, W. , Hoeksema, B. W. , Rachello‐Dolmen, P. G. , Moolenbeek, R. G. , Budiyanto, A. , Yahmantoro , Tuti, Y. , Giyanto , Draisma, S. , Prud'homme van Reine, W. F. , Hariyanto, R. , Gittenberger, A. , Rikoh, M. S. , & de Voogd, N. J. (2016). Variation in the composition of corals, fishes, sponges, echinoderms, ascidians, molluscs, foraminifera and macroalgae across a pronounced in‐to‐offshore environmental gradient in the Jakarta Bay‐Thousand Islands coral reef complex. Marine Pollution Bulletin, 110, 701–717. 10.1016/j.marpolbul.2016.04.042 27179997

[geb13513-bib-0030] Cleary, D. F. R. R. , & Renema, W. (2007). Relating species traits of foraminifera to environmental variables in the Spermonde Archipelago, Indonesia. Marine Ecology Progress Series, 334, 73–82. 10.3354/meps334073

[geb13513-bib-0031] Cornell, H. V. , & Harrison, S. P. (2014). What are species pools and when are they important? Annual Review of Ecology, Evolution, and Systematics, 45, 45–67. 10.1146/annurev-ecolsys-120213-091759

[geb13513-bib-0032] Cornwell, W. K. , & Ackerly, D. D. (2009). Community assembly and shifts in plant trait distributions across an environmental gradient in coastal California. Ecological Monographs, 79, 109–126. 10.1890/07-1134.1

[geb13513-bib-0033] Cottenie, K. (2005). Integrating environmental and spatial processes in ecological community dynamics. Ecology Letters, 8, 1175–1182. 10.1111/j.1461-0248.2005.00820.x 21352441

[geb13513-bib-0034] da Silva, P. G. , & Hernández, M. I. M. (2015). Scale‐dependence of processes structuring dung beetle metacommunities using functional diversity and community deconstruction approaches. PLoS One, 10, e0123030. 10.1371/journal.pone.0123030 25822150 PMC4378897

[geb13513-bib-0035] de Bello, F. , Botta‐Dukát, Z. , Lepš, J. , & Fibich, P. (2021). Towards a more balanced combination of multiple traits when computing functional differences between species. Methods in Ecology and Evolution, 12, 443–448. 10.1111/2041-210X.13537

[geb13513-bib-0036] Declerck, S. A. J. J. , Coronel, J. S. , Legendre, P. , & Brendonck, L. (2011). Scale dependency of processes structuring metacommunities of cladocerans in temporary pools of High‐Andes wetlands. Ecography, 34, 296–305. 10.1111/j.1600-0587.2010.06462.x

[geb13513-bib-0037] Dengler, J. , Wagner, V. , Dembicz, I. , García‐Mijangos, I. , Naqinezhad, A. , Boch, S. , Chiarucci, A. , Conradi, T. , Filibeck, G. , Guarino, R. , Janišová, M. , Steinbauer, M. J. , Acic, S. , Acosta, A. T. R. , Akasaka, M. , Allers, M. A. , Apostolova, I. , Axmanová, I. , Bakan, B. , … Biurrun, I. (2018). GrassPlot – A database of multi‐scale plant diversity in Palaearctic grasslands. Phytocoenologia, 48(3), 331–347. 10.1127/phyto/2018/0267

[geb13513-bib-0038] Díaz, A. M. , Alonso, M. L. S. , & Gutiérrez, M.‐R.‐V.‐A. (2007). Biological traits of stream macroinvertebrates from a semi‐arid catchment: Patterns along complex environmental gradients. Freshwater Biology, 53, 1–21. 10.1111/j.1365-2427.2007.01854.x

[geb13513-bib-0039] Drakare, S. , Lennon, J. J. , & Hillebrand, H. (2005). The imprint of the geographical, evolutionary and ecological context on species‐area relationships. Ecology Letters, 9, 215–227. 10.1111/j.1461-0248.2005.00848.x 16958886

[geb13513-bib-0040] Dziock, F. , Gerisch, M. , Siegert, M. , Hering, I. , Scholz, M. , & Ernst, R. (2011). Reproducing or dispersing? Using trait based habitat templet models to analyse Orthoptera response to flooding and land use. Agriculture, Ecosystems and Environment, 145, 85–94. 10.1016/j.agee.2011.07.015

[geb13513-bib-0041] Eallonardo, A. S. , Leopold, D. J. , Fridley, J. D. , & Stella, J. C. (2013). Salinity tolerance and the decoupling of resource axis plant traits. Journal of Vegetation Science, 24, 365–374. 10.1111/j.1654-1103.2012.01470.x

[geb13513-bib-0042] Edie, S. M. , Jablonski, D. , & Valentine, J. W. (2018). Contrasting responses of functional diversity to major losses in taxonomic diversity. Proceedings of the National Academy of Sciences of the United States of America, 115, 732–737. 10.1073/pnas.1717636115 29305556 PMC5789943

[geb13513-bib-0043] Farneda, F. Z. , Rocha, R. , López‐Baucells, A. , Groenenberg, M. , Silva, I. , Palmeirim, J. M. , Bobrowiec, P. E. D. D. , & Meyer, C. F. J. J. (2015). Trait‐related responses to habitat fragmentation in Amazonian bats. Journal of Applied Ecology, 52, 1381–1391. 10.1111/1365-2664.12490

[geb13513-bib-0134] Fontaneto, D. (2019). Long‐distance passive dispersal in microscopic aquatic animals. Movement Ecology, 7(1). 10.1186/s40462-019-0155-7 PMC6434837 30962931

[geb13513-bib-0044] Frenette‐Dussault, C. , Shipley, B. , Léger, J.‐F. , Meziane, D. , & Hingrat, Y. (2011). Functional structure of an arid steppe plant community reveals similarities with Grime’s C‐S‐R theory. Journal of Vegetation Science, 23, 208–222. 10.1111/j.1654-1103.2011.01350.x

[geb13513-bib-0045] Fried, G. , Kazakou, E. , & Gaba, S. (2012). Trajectories of weed communities explained by traits associated with species’ response to management practices. Agriculture, Ecosystems and Environment, 158, 147–155. 10.1016/j.agee.2012.06.005

[geb13513-bib-0046] Gallardo, B. , Gascón, S. , García, M. , & Comín, F. A. (2009). Testing the response of macroinvertebrate functional structure and biodiversity to flooding and confinement. Journal of Limnology, 68, 315–326. 10.4081/jlimnol.2009.315

[geb13513-bib-0047] Gazol, A. , Tamme, R. , Price, J. N. , Hiiesalu, I. , Laanisto, L. , & Pärtel, M. (2013). A negative heterogeneity–diversity relationship found in experimental grassland communities. Oecologia, 173, 545–555. 10.1007/s00442-013-2623-x 23468237

[geb13513-bib-0048] Gibb, H. , Muscat, D. , Binns, M. R. , Silvey, C. J. , Peters, R. A. , Warton, D. I. , & Andrew, N. R. (2015). Responses of foliage‐living spider assemblage composition and traits to a climatic gradient in Themeda grasslands. Austral Ecology, 40, 225–237.

[geb13513-bib-0049] Gómez‐Rodríguez, C. , & Baselga, A. (2018). Variation among European beetle taxa in patterns of distance decay of similarity suggests a major role of dispersal processes. Ecography, 41, 1825–1834. 10.1111/ecog.03693

[geb13513-bib-0050] Gonҫalves‐Souza, T. , Romero, G. Q. , Cottenie, K. , Gonçalves‐Souza, T. , Romero, G. Q. , & Cottenie, K. (2014). Metacommunity versus biogeography: A case study of two groups of neotropical vegetation‐dwelling arthropods. PLoS One, 9, 1–20. 10.1371/journal.pone.0115137 PMC4280172 25549332

[geb13513-bib-0051] Gossner, M. M. , Lewinsohn, T. M. , Kahl, T. , Grassein, F. , Boch, S. , Prati, D. , Birkhofer, K. , Renner, S. C. , Sikorski, J. , Wubet, T. , Arndt, H. , Baumgartner, V. , Blaser, S. , Blüthgen, N. , Börschig, C. , Buscot, F. , Diekötter, T. , Jorge, L. R. , Jung, K. , … Allan, E. (2016). Land‐use intensification causes multitrophic homogenization of grassland communities. Nature, 540, 266–269. 10.1038/nature20575 27919075

[geb13513-bib-0052] Gotelli, N. J. , & Graves, G. R. (1996). Null models in ecology. Smithsonian Institution Press.

[geb13513-bib-0053] Halpern, B. S. , Walbridge, S. , Selkoe, K. A. , Kappel, C. V. , Micheli, F. , D'Agrosa, C. , Bruno, J. F. , Casey, K. S. , Ebert, C. , Fox, H. E. , Fujita, R. , Heinemann, D. , Lenihan, H. S. , Madin, E. M. P. , Perry, M. T. , Selig, E. R. , Spalding, M. , Steneck, R. , & Watson, R. (2008). A global map of human impact on marine ecosystems. Science, 319, 948–952. 10.1126/science.1149345 18276889

[geb13513-bib-0054] Heim, N. A. , Payne, J. L. , Finnegan, S. , Knope, M. L. , Kowalewski, M. , Lyons, S. K. , McShea, D. W. , Novack‐Gottshall, P. M. , Smith, F. A. , & Wang, S. C. (2017). Hierarchical complexity and the size limits of life. Proceedings of the Royal Society of London. Series B: Biological Sciences, 284, 20171039. 10.1098/rspb.2017.1039 PMC5489738 28637850

[geb13513-bib-0055] Heino, J. , & Tolonen, K. T. (2017). Ecological drivers of multiple facets of beta diversity in a lentic macroinvertebrate metacommunity. Limnology and Oceanography, 62, 2431–2444. 10.1002/lno.10577

[geb13513-bib-0056] Hijmans, R. J. , Phillips, S. , Leathwick, J. , & Elith, J. (2017). dismo: species distribution modeling. https://CRAN.R‐project.org/package=dismo

[geb13513-bib-0057] Hillebrand, H. (2004). On the generality of the latitudinal diversity gradient. American Naturalist, 163, 192–211. 10.1086/381004 14970922

[geb13513-bib-0058] Hodgson, J. G. , Wilson, P. J. , Hunt, R. , Grime, J. P. , & Thompson, K. (1999). Allocating C‐S‐R plant functional types: A soft approach to a hard problem. Oikos, 85, 282. 10.2307/3546494

[geb13513-bib-0059] Hubbell, S. P. (2001). The unified neutral theory of biodiversity and biogeography. Princeton University Press. 10.1016/j.tree.2011.03.024 21561679

[geb13513-bib-0060] Hutchinson, G. E. (1957). Concluding remarks. Cold Spring Harbor Symposia on Quantitative Biology, 22, 415–427. 10.1101/SQB.1957.022.01.039

[geb13513-bib-0061] IPBES . (2019). Global assessment report on biodiversity and ecosystem services of the Intergovernmental Science‐Policy Platform on Biodiversity and Ecosystem Services. E. S. Brondizio , J. Settele , S. Díaz , & H. T. Ngo (Eds.). IPBES Secretariat.

[geb13513-bib-0062] Jamil, T. , Ozinga, W. A. , Kleyer, M. , & ter Braak, C. J. F. F. (2013). Selecting traits that explain species‐environment relationships: A generalized linear mixed model approach. Journal of Vegetation Science, 24, 988–1000. 10.1111/j.1654-1103.2012.12036.x

[geb13513-bib-0063] Jamoneau, A. , Chabrerie, O. , Closset‐Kopp, D. , & Decocq, G. (2012). Fragmentation alters beta‐diversity patterns of habitat specialists within forest metacommunities. Ecography, 35, 124–133. 10.1111/j.1600-0587.2011.06900.x

[geb13513-bib-0064] Jarzyna, M. A. , & Jetz, W. (2018) Taxonomic and functional diversity change is scale dependent. Nature Communications, 9(1), 1–8. 10.1038/s41467-018-04889-z PMC6028399 29967400

[geb13513-bib-0065] Jarzyna, M. A. , Quintero, I. , & Jetz, W. (2021). Global functional and phylogenetic structure of avian assemblages across elevation and latitude. Ecology Letters, 24, 196–207. 10.1111/ele.13631 33124188

[geb13513-bib-0066] Jeliazkov, A. , Chiron, F. , Garnier, J. , Besnard, A. , Silvestre, M. , & Jiguet, F. (2014). Level‐dependence of the relationships between amphibian biodiversity and environment in pond systems within an intensive agricultural landscape. Hydrobiologia, 723, 7–23. 10.1007/s10750-013-1503-z

[geb13513-bib-0067] Jeliazkov, A. , Mijatovic, D. , Chantepie, S. , Andrew, N. , Arlettaz, R. , Barbaro, L. , Barsoum, N. , Bartonova, A. , Belskaya, E. , Bonada, N. , Brind’Amour, A. , Carvalho, R. , Castro, H. , Chmura, D. , Choler, P. , Chong‐Seng, K. , Cleary, D. , Cormont, A. , Cornwell, W. , … Chase, J. M. (2020). A global database for metacommunity ecology, integrating species, traits, environment and space. Scientific Data, 7, 6. 10.1038/s41597-019-0344-7 31913312 PMC6949231

[geb13513-bib-0068] Jenkins, D. G. , Brescacin, C. R. , Duxbury, C. V. , Elliott, J. A. , Evans, J. A. , Grablow, K. R. , Hillegass, M. , Lyon, B. N. , Metzger, G. A. , Olandese, M. L. , Pepe, D. , Silvers, G. A. , Suresch, H. N. , Thompson, T. N. , Trexler, C. M. , Williams, G. E. , Williams, N. C. , & Williams, S. E. (2007). Does size matter for dispersal distance? Global Ecology and Biogeography, 16, 415–425. 10.1111/j.1466-8238.2007.00312.x

[geb13513-bib-0069] Kattge, J. , Díaz, S. , Lavorel, S. , Prentice, I. C. , Leadley, P. , Bönisch, G. , Garnier, E. , Westoby, M. , Reich, P. B. , Wright, I. J. , Cornelissen, J. H. C. , Violle, C. , Harrison, S. P. , Van BODEGOM, P. M. , Reichstein, M. , Enquist, B. J. , Soudzilovskaia, N. A. , Ackerly, D. D. , Anand, M. , … Wirth, C. (2011). TRY ‐ a global database of plant traits. Global Change Biology, 17, 2905–2935. 10.1111/j.1365-2486.2011.02451.x

[geb13513-bib-0070] Kraft, N. J. B. , Comita, L. S. , Chase, J. M. , Sanders, N. J. , Swenson, N. G. , Crist, T. O. , Stegen, J. C. , Vellend, M. , Boyle, B. , Anderson, M. J. , Cornell, H. V. , Davies, K. F. , Freestone, A. L. , Inouye, B. D. , Harrison, S. P. , & Myers, J. A. (2011). Disentangling the drivers of β diversity along latitudinal and elevational gradients. Science, 333, 1755–1758. 10.1126/science.1208584 21940897

[geb13513-bib-0071] Krasnov, B. R. , Shenbrot, G. I. , Khokhlova, I. S. , Stanko, M. , Morand, S. , & Mouillot, D. (2015). Assembly rules of ectoparasite communities across scales: Combining patterns of abiotic factors, host composition, geographic space, phylogeny and traits. Ecography, 38, 184–197. 10.1111/ecog.00915

[geb13513-bib-0072] Kruk, C. , Segura, A. M. , Costa, L. S. , Lacerot, G. , Kosten, S. , Peeters, E. T. H. M. , Huszar, V. L. M. , Mazzeo, N. , & Scheffer, M. (2017). Functional redundancy increases towards the tropics in lake phytoplankton. Journal of Plankton Research, 39, 518–530.

[geb13513-bib-0073] Laanisto, L. , Tamme, R. , Hiiesalu, I. , Szava‐Kovats, R. , Gazol, A. , & Pärtel, M. (2012). Microfragmentation concept explains non‐positive environmental heterogeneity–diversity relationships. Oecologia, 171, 217–226. 10.1007/s00442-012-2398-5 22752212

[geb13513-bib-0074] Lamanna, C. , Blonder, B. , Violle, C. , Kraft, N. J. B. B. , Sandel, B. , Šímová, I. , Donoghue, J. C. 2nd , Svenning, J.‐C.‐C. , McGill, B. J. , Boyle, B. , Buzzard, V. , Dolins, S. , Jørgensen, P. M. , Marcuse‐Kubitza, A. , Morueta‐Holme, N. , Peet, R. K. , Piel, W. H. , Regetz, J. , Schildhauer, M. , … Enquist, B. J. (2014). Functional trait space and the latitudinal diversity gradient. Proceedings of the National Academy of Sciences of the United States of America, 111, 13745–13750. 10.1073/pnas.1317722111 25225365 PMC4183280

[geb13513-bib-0075] Leibold, M. A. , & McPeek, M. A. (2006). Coexistence of the niche and neutral perspectives in community ecology. Ecology, 87, 1399–1410. 16869414 10.1890/0012-9658(2006)87[1399:cotnan]2.0.co;2

[geb13513-bib-0076] Lowe, E. C. , Threlfall, C. G. , Wilder, S. M. , & Hochuli, D. F. (2018). Environmental drivers of spider community composition at multiple scales along an urban gradient. Biodiversity and Conservation, 27, 829–852. 10.1007/s10531-017-1466-x

[geb13513-bib-0077] Mammola, S. , & Cardoso, P. (2020). Functional diversity metrics using kernel density n‐dimensional hypervolumes. Methods in Ecology and Evolution, 11, 986–995.

[geb13513-bib-0078] Mammola, S. , Carmona, C. P. , Guillerme, T. , & Cardoso, P. (2021). Concepts and applications in functional diversity. Functional Ecology, 35(9), 1869–1885.

[geb13513-bib-0079] Martin, K. , Schmidt, K. , Toseland, A. , Boulton, C. A. , Barry, K. , Beszteri, B. , Brussaard, C. P. D. , Clum, A. , Daum, C. G. , Eloe‐Fadrosh, E. , Fong, A. , Foster, B. , Foster, B. , Ginzburg, M. , Huntemann, M. , Ivanova, N. N. , Kyrpides, N. C. , Lindquist, E. , Mukherjee, S. , … Mock, T. (2021) The biogeographic differentiation of algal microbiomes in the upper ocean from pole to pole. Nature Communications, 12(1), 1–15. 10.1038/s41467-021-25646-9 PMC8446083 34531387

[geb13513-bib-0080] Martínez, A. , García‐Gómez, G. , García‐Herrero, Á. , Sánchez, N. , Pardos, F. , Izquierdo‐Muñoz, A. , Fontaneto, D. , & Mammola, S. (2021). Habitat differences filter functional diversity of low dispersive microscopic animals (Acari, Halacaridae). Hydrobiologia, 848, 2681–2698. 10.1007/s10750-021-04586-x

[geb13513-bib-0081] McGill, B. J. , Enquist, B. J. , Weiher, E. , & Westoby, M. (2006). Rebuilding community ecology from functional traits. Trends in Ecology and Evolution, 21, 178–185. 10.1016/j.tree.2006.02.002 16701083

[geb13513-bib-0082] Meffert, P. J. , & Dziock, F. (2013). The influence of urbanisation on diversity and trait composition of birds. Landscape Ecology, 28, 943–957. 10.1007/s10980-013-9867-z

[geb13513-bib-0083] Meynard, C. N. , Devictor, V. , Mouillot, D. , Thuiller, W. , Jiguet, F. , & Mouquet, N. (2011). Beyond taxonomic diversity patterns: How do α, β and γ components of bird functional and phylogenetic diversity respond to environmental gradients across France? Global Ecology and Biogeography, 20, 893–903. 10.1111/j.1466-8238.2010.00647.x

[geb13513-bib-0084] Millar, R. B. , Anderson, M. J. , & Tolimieri, N. (2011). Much ado about nothings: Using zero similarity points in distance‐decay curves. Ecology, 92(9), 1717–1722. 10.1890/11-0029.1 21939067

[geb13513-bib-0085] Mori, A. S. , Isbell, F. , & Seidl, R. (2018). β‐diversity, community assembly, and ecosystem functioning. Trends in Ecology and Evolution, 33, 549–564. 10.1016/j.tree.2018.04.012 29807839 PMC7612777

[geb13513-bib-0086] Morlon, H. , Chuyong, G. , Condit, R. , Hubbell, S. P. , Kenfack, D. , Thomas, D. , Valencia, R. , & Green, J. L. (2008). A general framework for the distance‐decay of similarity in ecological communities. Ecology Letters, 11, 904–917. 10.1111/j.1461-0248.2008.01202.x 18494792 PMC2613237

[geb13513-bib-0087] Mouillot, D. , Graham, N. A. J. J. , Villéger, S. , Mason, N. W. H. H. , & Bellwood, D. R. (2013). A functional approach reveals community responses to disturbances. Trends in Ecology and Evolution, 28, 167–177. 10.1016/j.tree.2012.10.004 23141923

[geb13513-bib-0088] Mouillot, D. , Villéger, S. , Parravicini, V. , Kulbicki, M. , Arias‐González, J. E. , Bender, M. , Chabanet, P. , Floeter, S. R. , Friedlander, A. , Vigliola, L. , & Bellwood, D. R. (2014). Functional over‐redundancy and high functional vulnerability in global fish faunas on tropical reefs. Proceedings of the National Academy of Sciences of the United States of America, 111, 13757–13762. 10.1073/pnas.1317625111 25225388 PMC4183327

[geb13513-bib-0089] Mouquet, N. , & Loreau, M. (2003). Community patterns in source‐sink metacommunities. The American Naturalist, 162, 544–557. 10.1086/378857 14618534

[geb13513-bib-0090] Múrria, C. , Iturrarte, G. , & Gutiérrez‐Cánovas, C. (2020). A trait space at an overarching scale yields more conclusive macroecological patterns of functional diversity. Global Ecology and Biogeography, 29, 1729–1742. 10.1111/geb.13146

[geb13513-bib-0091] Nekola, J. C. , & McGill, B. J. (2014). Scale dependency in the functional form of the distance decay relationship. Ecography, 37, 309–320. 10.1111/j.1600-0587.2013.00407.x

[geb13513-bib-0092] Nekola, J. C. , & White, P. S. (1999). The distance decay of similarity in biogeography and ecology. Journal of Biogeography, 26, 867–878. 10.1046/j.1365-2699.1999.00305.x

[geb13513-bib-0133] Oksanen, J. , Blanchet, F. G. , Friendly, M. , Kindt, R. , Legendre, P. , McGlinn, D. , Minchin, P. R. , O’Hara, R. B. , Simpson, G. L. , Solymos, P. , Stevens, M. H. H. , Szoecs, E. , & Wagner, H. (2019). vegan: Community Ecology Package. https://CRAN.R‐project.org/package=vegan

[geb13513-bib-0093] Pakeman, R. J. (2011). Multivariate identification of plant functional response and effect traits in an agricultural landscape. Ecology, 92, 1353–1365. 10.1890/10-1728.1 21797163

[geb13513-bib-0094] Palmer, M. W. , & White, P. S. (1994). Scale dependence and the species‐area relationship. The American Naturalist, 144(5), 717–740. 10.1086/285704

[geb13513-bib-0095] Penone, C. , Weinstein, B. G. , Graham, C. H. , Brooks, T. M. , Rondinini, C. , Hedges, S. B. , Davidson, A. D. , & Costa, G. C. (2016). Global mammal beta diversity shows parallel assemblage structure in similar but isolated environments. Proceedings of the Royal Society of London. Series B: Biological Sciences, 283, 1–9. 10.1098/rspb.2016.1028 PMC5013794 27559061

[geb13513-bib-0096] Peters, R. H. (1983). The ecological implications of body size. Cambridge University Press.

[geb13513-bib-0097] Pianka, E. R. (1966). Latitudinal gradients in species diversity: A review of concepts. The American Naturalist, 100, 33–46. 10.1086/282398

[geb13513-bib-0098] Qian, H. , Badgley, C. , & Fox, D. L. (2009). The latitudinal gradient of beta diversity in relation to climate and topography for mammals in North America. Global Ecology and Biogeography, 18, 111–122. 10.1111/j.1466-8238.2008.00415.x

[geb13513-bib-0099] Questad, E. J. , & Foster, B. L. (2008). Coexistence through spatio‐temporal heterogeneity and species sorting in grassland plant communities. Ecology Letters, 11, 717–726. 10.1111/j.1461-0248.2008.01186.x 18445035

[geb13513-bib-1000] Rachello‐Dolmen, P. G. , & Cleary, D. F. R. (2007). Relating coral species traits to environmental conditions in the Jakarta Bay/Pulau Seribu reef system, Indonesia. Estuarine, Coastal and Shelf Science, 73(3–4), 816–826. 10.1016/j.ecss.2007.03.017

[geb13513-bib-0100] Raevel, V. , Violle, C. , & Munoz, F. (2012). Mechanisms of ecological succession: Insights from plant functional strategies. Oikos, 121, 1761–1770. 10.1111/j.1600-0706.2012.20261.x

[geb13513-bib-0101] Ribera, I. , Dolédec, S. , Downie, I. S. , & Foster, G. N. (2001). Effect of land disturbance and stress on species traits of ground beetle assemblages. Ecology, 82, 1112–1129.

[geb13513-bib-0102] Robinson, N. , Kadlec, T. , Bowers, M. D. , & Guralnick, R. P. (2014). Integrating species traits and habitat characteristics into models of butterfly diversity in a fragmented ecosystem. Ecological Modelling, 281, 15–25. 10.1016/j.ecolmodel.2014.01.022

[geb13513-bib-0103] Robroek, B. J. M. M. , Jassey, V. E. J. J. , Payne, R. J. , Martí, M. , Bragazza, L. , Bleeker, A. , Buttler, A. , Caporn, S. J. M. M. , Dise, N. B. , Kattge, J. , Zając, K. , Svensson, B. H. , van Ruijven, J. , Verhoeven, J. T. A. A. , Zajac, K. , Svensson, B. H. , van Ruijven, J. , & Verhoeven, J. T. A. A. (2017). Taxonomic and functional turnover are decoupled in European peat bogs. Nature Communications, 8, 1161. 10.1038/s41467-017-01350-5 PMC5660083 29079831

[geb13513-bib-0104] Rohde, K. (1996). Rapoport’s Rule is a local phenomenon and cannot explain latitudinal gradients in species diversity. Biodiversity Letters, 3, 10–13. 10.2307/2999704

[geb13513-bib-0105] Shurin, J. B. , Gruner, D. S. , & Hillebrand, H. (2006). Review All wet or dried up? Real differences between aquatic and terrestrial food webs. Proceedings of the Royal Society of London. Series B: Biological Sciences, 273, 1–9. 10.1098/rspb.2005.3377 PMC1560001 16519227

[geb13513-bib-0106] Siefert, A. , Ravenscroft, C. , Weiser, M. D. , & Swenson, N. G. (2013). Functional beta‐diversity patterns reveal deterministic community assembly processes in eastern North American trees. Global Ecology and Biogeography, 22, 682–691. 10.1111/geb.12030

[geb13513-bib-0107] Soininen, J. (2010). Species turnover along abiotic and biotic gradients: patterns in space equal patterns in time? BioScience, 60, 433–439. 10.1525/bio.2010.60.6.7

[geb13513-bib-0108] Soininen, J. , & Hillebrand, H. (2007). Disentangling distance decay of similarity from richness gradients: Response to Baselga (2007). Ecography, 30, 842–844. 10.1111/j.2007.0906-7590.05387.x

[geb13513-bib-0109] Soininen, J. , Jamoneau, A. , Rosebery, J. , & Passy, S. I. (2016). Global patterns and drivers of species and trait composition in diatoms. Global Ecology and Biogeography, 25, 940–950. 10.1111/geb.12452

[geb13513-bib-0110] Soininen, J. , Lennon, J. J. , & Hillebrand, H. (2007). A multivariate analysis of beta diversity across organisms and environments. Ecology, 88, 2830–2838. 10.1890/06-1730.1 18051652

[geb13513-bib-0111] Soininen, J. , McDonald, R. , & Hillebrand, H. (2007). The distance decay of similarity in ecological communities. Ecography, 30, 3–12. 10.1111/j.0906-7590.2007.04817.x

[geb13513-bib-0112] Sokol, E. R. , Benfield, E. F. , Belden, L. K. , & Maurice Valett, H. (2011). The assembly of ecological communities inferred from taxonomic and functional composition. The American Naturalist, 177, 630–644. 10.1086/659625 21508609

[geb13513-bib-0113] Spasojevic, M. J. , Yablon, E. A. , Oberle, B. , & Myers, J. A. (2014). Ontogenetic trait variation influences tree community assembly across environmental gradients. Ecosphere, 5, 1–20. 10.1890/ES14-000159.1

[geb13513-bib-0114] Stegen, J. C. , Lin, X. , Fredrickson, J. K. , Chen, X. , Kennedy, D. W. , Murray, C. J. , Rockhold, M. L. , & Konopka, A. (2013). Quantifying community assembly processes and identifying features that impose them. The ISME Journal, 7, 2069–2079. 10.1038/ismej.2013.93 23739053 PMC3806266

[geb13513-bib-0115] Steinbauer, M. J. , Dolos, K. , Reineking, B. , & Beierkuhnlein, C. (2012). Current measures for distance decay in similarity of species composition are influenced by study extent and grain size. Global Ecology and Biogeography, 21, 1203–1212. 10.1111/j.1466-8238.2012.00772.x

[geb13513-bib-0135] Stevens, G. C. (1989). The latitudinal gradient in geographical range: How so many species coexist in the tropics. The American Naturalist, 133(2), 240–256. 10.1086/284913

[geb13513-bib-0117] Swenson, N. G. , Anglada‐Cordero, P. , & Barone, J. A. (2011). Deterministic tropical tree community turnover: Evidence from patterns of functional beta diversity along an elevational gradient. Proceedings of the Royal Society of London. Series B: Biological Sciences, 278, 877–884. 10.1098/rspb.2010.1369 PMC3049044 20861048

[geb13513-bib-0118] Teittinen, A. , & Virta, L. (2021). Exploring multiple aspects of taxonomic and functional diversity in microphytobenthic communities: effects of environmental gradients and temporal changes. Frontiers in Microbiology, 12, 668993. 10.3389/fmicb.2021.668993 34093487 PMC8175668

[geb13513-bib-0119] Terborgh, J. (1973). On the notion of favorableness in plant ecology. The American Naturalist, 107, 481–501. 10.1086/282852

[geb13513-bib-0120] Titley, M. A. , Snaddon, J. L. , & Turner, E. C. (2017). Scientific research on animal biodiversity is systematically biased towards vertebrates and temperate regions. PLoS One, 12, e0189577. 10.1371/journal.pone.0189577 29240835 PMC5730207

[geb13513-bib-0121] Triantis, K. A. , Guilhaumon, F. , & Whittaker, R. J. (2011). The island species‐area relationship: Biology and statistics. Journal of Biogeography, 39, 215–231. 10.1111/j.1365-2699.2011.02652.x

[geb13513-bib-0122] van Klink, R. , Boch, S. , Buri, P. , Rieder, N. S. , Humbert, J.‐Y.‐Y. , & Arlettaz, R. (2017). No detrimental effects of delayed mowing or uncut grass refuges on plant and bryophyte community structure and phytomass production in low‐intensity hay meadows. Basic and Applied Ecology, 20, 1–9. 10.1016/j.baae.2017.02.003

[geb13513-bib-0123] Vavrek, M. J. (2011). fossil: Palaeoecological and palaeogeographical analysis tools. Palaeontologia Electronica, 14, 16.

[geb13513-bib-0124] Venter, O. , Sanderson, E. W. , Magrach, A. , Allan, J. R. , Beher, J. , Jones, K. R. , Possingham, H. P. , Laurance, W. F. , Wood, P. , Fekete, B. M. , Levy, M. A. , & Watson, J. E. M. (2016). Sixteen years of change in the global terrestrial human footprint and implications for biodiversity conservation. Nature Communications, 7(1), 1–11. 10.1038/ncomms12558 PMC4996975 27552116

[geb13513-bib-0125] Villéger, S. , Grenouillet, G. , & Brosse, S. (2013). Decomposing functional β‐diversity reveals that low functional β‐diversity is driven by low functional turnover in European fish assemblages. Global Ecology and Biogeography, 22, 671–681. 10.1111/geb.12021

[geb13513-bib-0126] Villéger, S. , Mason, N. W. H. H. , & Mouillot, D. (2008). New multidimensional functional diversity indices for a multifaceted framework in functional ecology. Ecology, 89, 2290–2301. 10.1890/07-1206.1 18724739

[geb13513-bib-0127] Villéger, S. , Ramos Miranda, J. , Flores Hernandez, D. , & Mouillot, D. (2012). Low functional β‐diversity despite high taxonomic β‐diversity among tropical estuarine fish communities. PLoS One, 7, e40679. 10.1371/journal.pone.0040679 22792395 PMC3392234

[geb13513-bib-0128] Weinstein, B. G. , Tinoco, B. , Parra, J. L. , Brown, L. M. , McGuire, J. A. , Stiles, F. G. , & Graham, C. H. (2014). Taxonomic, phylogenetic, and trait beta diversity in south american hummingbirds. The American Naturalist, 184, 211–224. 10.1086/676991 25058281

[geb13513-bib-0132] Wickham, H. , Averick, M. , Bryan, J. , Chang, W. , McGowan, L. , François, R. , Grolemund, G. , Hayes, A. , Henry, L. , Hester, J. , Kuhn, M. , Pedersen, T. , Miller, E. , Bache, S. , Müller, K. , Ooms, J. , Robinson, D. , Seidel, D. , Spinu, V. , … Yutani, H. (2019). Welcome to the Tidyverse. Journal of Open Source Software, 4(43), 1686. 10.21105/joss.01686

[geb13513-bib-0129] Zagmajster, M. , Eme, D. , Fišer, C. , Galassi, D. , Marmonier, P. , Stoch, F. , Cornu, J. F. , & Malard, F. (2014). Geographic variation in range size and beta diversity of groundwater crustaceans: Insights from habitats with low thermal seasonality. Global Ecology and Biogeography, 23, 1135–1145. 10.1111/geb.12200

[geb13513-bib-0130] Zhu, L. , Fu, B. , Zhu, H. , Wang, C. , Jiao, L. , & Zhou, J. (2017). Trait choice profoundly affected the ecological conclusions drawn from functional diversity measures. Scientific Reports, 7, 3643. 10.1038/s41598-017-03812-8 28623286 PMC5473860

