# Supplementary material for: Distance decay 2.0 – A global synthesis of taxonomic and functional turnover in ecological communities
Source: Glob Ecol Biogeogr. 2022 May 12;31(7):1399–421. doi: 10.1111/geb.13513 (PMC9322010; doi:10.1111/geb.13513)
Supplement: Supplementary file 2 — Appendix S2 [file GEB-31-1399-s003.docx]

Appendix to: Distance decay 2.0 – a global synthesis of taxonomic and functional decay in ecological communities

*Appendix S2*

**Data curation.** For each dataset, we removed the sites with less than two observed species, and the species with lower than three traits or trait groups. Trait data included ordered, categorical and continuous traits. Environmental variables were log-transformed (Log_10_) to approximate normality (except for e.g., temperature, pH and variables given as eigenvectors), and the environmental variables showing strong inter-correlations (pairwise r_p_ < 0.7) were excluded from further analyses (Leathwick *et al.*, 2006). The final set of environmental variables were standardized the environmental variables to µ = 0 and σ = 1. Spatial coordinates were converted to the World Geodetic System 1984 (WGS84) datum and geographic coordinate system and expressed in decimal degrees with an accuracy up to five decimals.

**Partition of similarities.** Traditionally, β-diversity has been measured by comparing the matching/mismatching components of pairs of communities (Legendre, 2014). For an occurrence-based β-diversity, these components are the number of features, e.g. species, (a) shared by both communities, (b) present in the first community only, (c) present in the second community only and (d) absent in both communities but present elsewhere in the region. For an abundance-based β-diversity, we use the upper-case letter (A) to designate the sum of the abundances of the multiple features; (B) to represent the sum of abundances at the first community minus A; and (C) as the sum of abundances at the second community minus A. Therefore, the upper and lower-case components are analogous to each other and represent both the intersections and community-specific features of the studied sites. β-diversity can thus be calculated and partitioned into total, replacement, and richness differences components by applying some dissimilarity index (Baselga & Leprieur, 2015) such as Sørensen index (Sørensen, 1948) on occurrence data or equivalently the percentage differences index (Odum, 1950) on abundance data as shown in Table S1.

**Table S1** Equations used for the estimation of total similarities and replacement and richness differences components using occurrence and abundance information.

|  | Ocurrence data | Abundance data |
| --- | --- | --- |
| Total | $1-(b+c)/(2a+b+c)$ | $1-(B+C)/(2A+B+C)$ |
| Replacement | $1-(2 \times min(b,c))/(2a+b+c)$ | $1-(2 \times min(B,C))/(2A+B+C)$ |
| Richness differences | $1-(\vert b-c\vert)/(2a+b+c)$ | $1-(\vert B-C\vert)/(2A+B+C)$ |

**Boosted regression trees.** BRT is a regression modelling technique able to fit nonlinear relationships between predictor and response variables, including interaction among variables by using a boosting strategy to combine results from a large number (usually thousands) of simple regression tree models (Friedman, 2001). Our BRT outputs included graphs of the shapes of relationships between predictors and the response variable (e.g., linear, curvilinear, and sigmoidal response shapes) and a relative importance of predictor variables. ﻿We also plotted a LOESS line on these plots to allow for easy visualization of the central tendency of the predicted values. Relative importance is constructed by counting the number of times a variable is selected for splitting in each tree, weighted by the squared improvement of the model because of each split, and averaged over all tree (Friedman, 2001; Elith *et al.*, 2008; Hastie *et al.*, 2009) .We performed a 50–50 cross-validation procedure and estimated the model performance ($D^{2}= \frac{{Deviance}_{Total}-{Deviance}_{Cross-validation}}{{Deviance}_{Total}}$) following Leathwick et al.(2006).

While BRT can account for interactions by increasing the number of nodes (higher value in the *tree complexity* parameter), it does not directly provide information on the nature and magnitude of fitted interactions. Therefore, we used the function *gbm.interaction* that creates a temporary grid of variables representing combinations of values at fixed intervals along each of their ranges for each pair of predictors. Hence, predictions are formed on the linear predictor scale for the grid, while all other variables are set to their respective means. A linear model is used to relate these temporary predictions to the two marginal predictors, fitting the latter as factors. The residual variance in this linear model indicates the relative strength of interaction fitted by BRT, with a residual variance of zero indicating that no interaction effects are fitted (Elith *et al*., 2008).

**Sensitivity analysis.**

Figure S2.1. Sensitivity analysis ran with 100%, 90%, 70%, 50% of the data on taxonomic similarities along spatial gradients. The models were run with all the predictors, but only a few predictors are shown for simplicity. The figure shows that while the magnitude of the effect reduces with the subsets of data, main patterns are robust to differences in sample size.


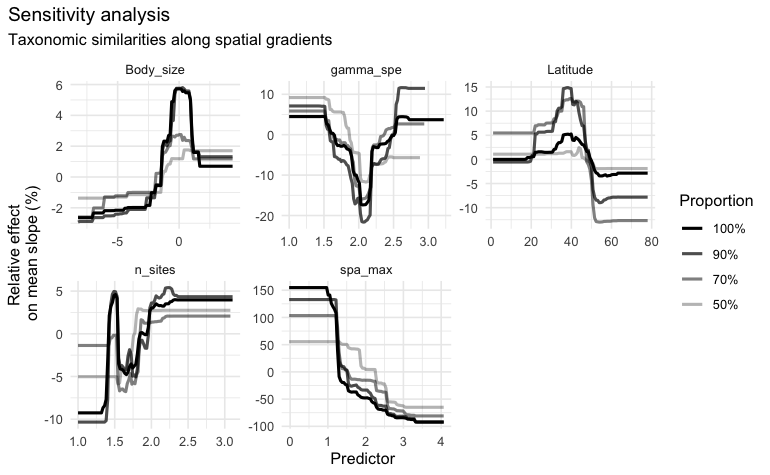


Figure S2.2. Sensitivity analysis ran with 100%, 90%, 70%, 50% of the data on functional similarities along spatial gradients. The models were run with all the predictors, but only a few predictors are shown for simplicity. The figure shows that while the magnitude of the effect reduces with the subsets of data, main patterns are robust to differences in sample size.


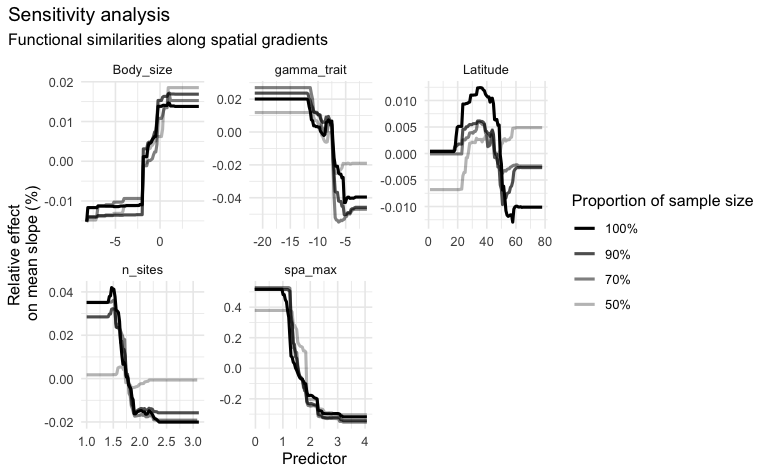


**References**

Baselga, A. & Leprieur, F. (2015) Comparing methods to separate components of beta diversity. *Methods in Ecology and Evolution*, **6**, 1069–1079.

Elith, J., Leathwick, J.R. & Hastie, T. (2008) A working guide to boosted regression trees. *Journal of Animal Ecology*, **77**, 802–813.

Friedman, J.H. (2001) Greedy function approximation: a gradient boosting machine. *Annals of statistics*, 1189–1232.

Hastie, T., Tibshirani, R. & Friedman, J. (2009) *The elements of statistical learning: data mining, inference, and prediction*, Springer Science & Business Media.

Leathwick, J.R., Elith, J. & Hastie, T. (2006) Comparative performance of generalized additive models and multivariate adaptive regression splines for statistical modelling of species distributions. *Ecological Modelling*, **199**, 188–196.

Legendre, P. (2014) Interpreting the replacement and richness difference components of beta diversity. *Global Ecology and Biogeography*, **23**, 1324–1334.

Odum, E.P. (1950) Bird Populations of the Highlands (North Carolina) Plateau in Relation to Plant Succession and Avian Invasion. *Ecology*, **31**.

Sørensen, T.A. (1948) A method of establishing groups of equal amplitude in plant sociology based on similarity of species content, and its application to analyses of the vegetation on Danish commons. *Kongelige Danske Videnskabernes Selskabs Biologiske Skrifter*, **5**.
