# Supplementary material for: Distance decay 2.0 – A global synthesis of taxonomic and functional turnover in ecological communities
Source: Glob Ecol Biogeogr. 2022 May 12;31(7):1399–421. doi: 10.1111/geb.13513 (PMC9322010; doi:10.1111/geb.13513)
Supplement: Supplementary file 4 — Appendix S4 [file GEB-31-1399-s005.docx]

Appendix to: Distance decay 2.0 – a global synthesis of taxonomic and functional decay in ecological communities

*Appendix S4*

**Results of occurrence-based replacement and richness differences components**

Organismal variables and dataset features

Actively dispersed taxa typically had the flattest slopes of replacement and richness differences along spatial and environmental distances (Fig. S4.9–S4.10). Slopes generally increased with body size, except for the richness differences component along space that showed a decrease (Fig. S4.9–S4.10). Replacement was highest in datasets with few species while richness differences was highest in the richest datasets (Fig. S4.9–S4.10). Higher functional γ-diversity resulted in flatter slopes of both replacement and richness differences, except for replacement along environment which showed a U-shaped relationship along environmental distances (Fig. S4.9–S4.10). Datasets with a few sites had flattest taxonomic replacement and steepest functional replacement (Fig. S4.9–S4.10). The number of environmental variables in the datasets generally increased slope values (Fig. S4.9–S4.10).


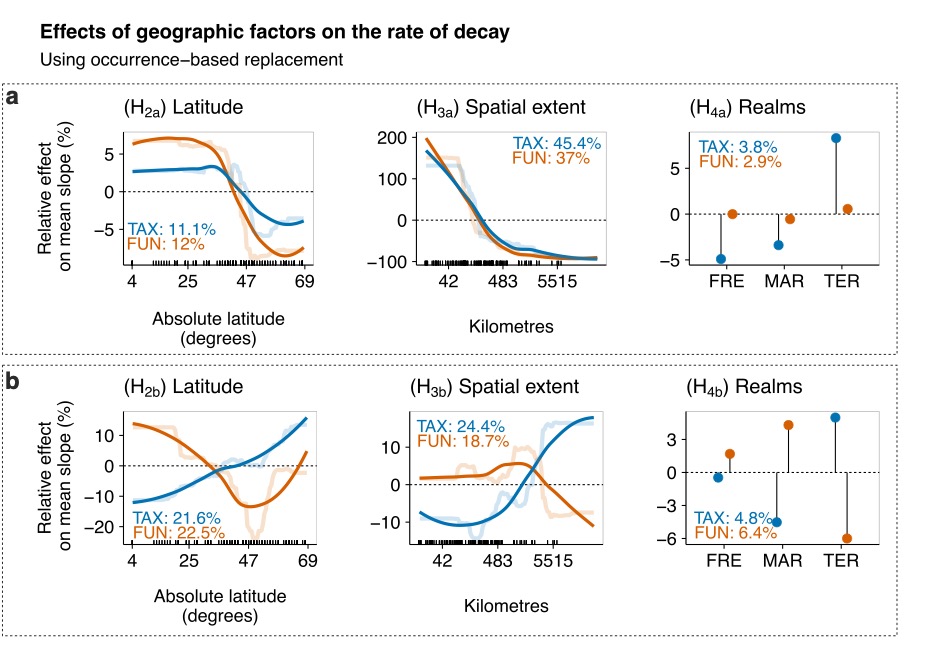


Figure S4.7. Relative effects (%) of geographic factors on the rate of decay along spatial (a) and environmental (b) distance decay of the replacement component of taxonomic (TAX - blue) and functional (FUN - orange) similarities using occurrence data across datasets. Partial dependence plots show the effects of a predictor variable on the response variable after accounting for the average effects of all other variables in the model. Positive values indicate an increase in the rate of decay (steeper slopes) compared to the mean rate whereas negative values indicate a decrease in the rate of decay (flatter slopes) compared to the mean rate. Semi-transparent lines represent the actual predicted effects; solid lines represent LOESS fits to predicted values from BRT. We show here only the variables related to the specific hypotheses, i.e., latitude, spatial extent, and realms (FRE = Freshwater, TER = Terrestrial, MAR = Marine).


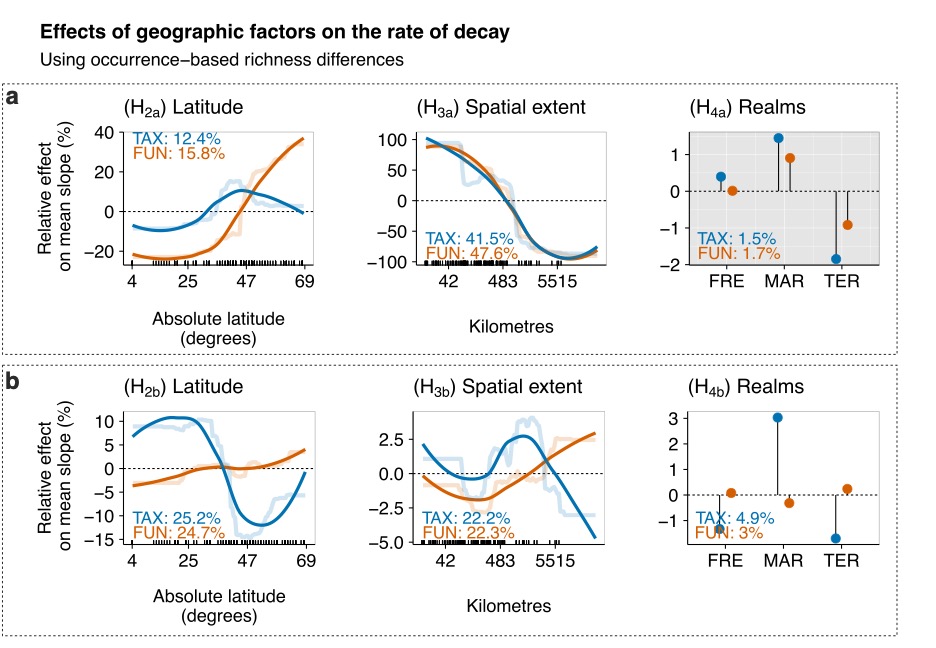


Figure S4.8. Relative effects (%) of geographic factors on the rate of decay along spatial (a) and environmental (b) distance decay of the richness differences component of taxonomic (TAX - blue) and functional (FUN - orange) similarities using occurrence data across datasets. Partial dependence plots show the effects of a predictor variable on the response variable after accounting for the average effects of all other variables in the model. Positive values indicate an increase in the rate of decay (steeper slopes) compared to the mean rate whereas negative values indicate a decrease in the rate of decay (flatter slopes) compared to the mean rate. Semi-transparent lines represent the actual predicted effects; solid lines represent LOESS fits to predicted values from BRT. We show here only the variables related to the specific hypotheses, i.e., latitude, spatial extent, and realms (FRE = Freshwater, TER = Terrestrial, MAR = Marine).


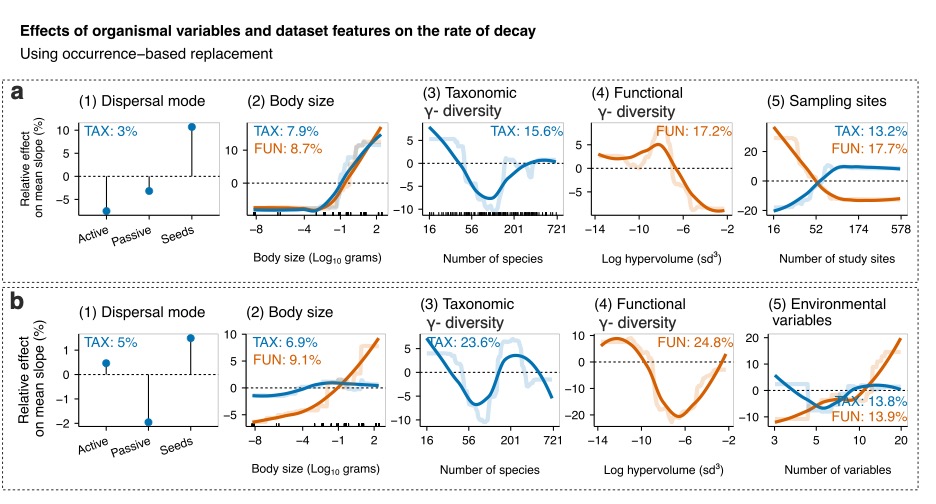


Figure S4.9. Relative effects (%) of organismal variables and dataset features on the rate of decay along spatial (a) and environmental (b) distance considering the replacement component of taxonomic (blue lines) and functional (orange lines) similarities using occurrence data across datasets. Partial dependence plots show the effects of a predictor variable on the response variable after accounting for the average effects of all other variables in the model. Positive values indicate an increase in the rate of decay (steeper slopes) compared to the mean rate whereas negative values indicate a decrease in the rate of decay (flatter slopes) compared to the mean rate. Semi-transparent lines represent the actual predicted effects; solid lines represent LOESS fits to predicted values from BRT. We show here the organismal variables and the variables related to the dataset features.


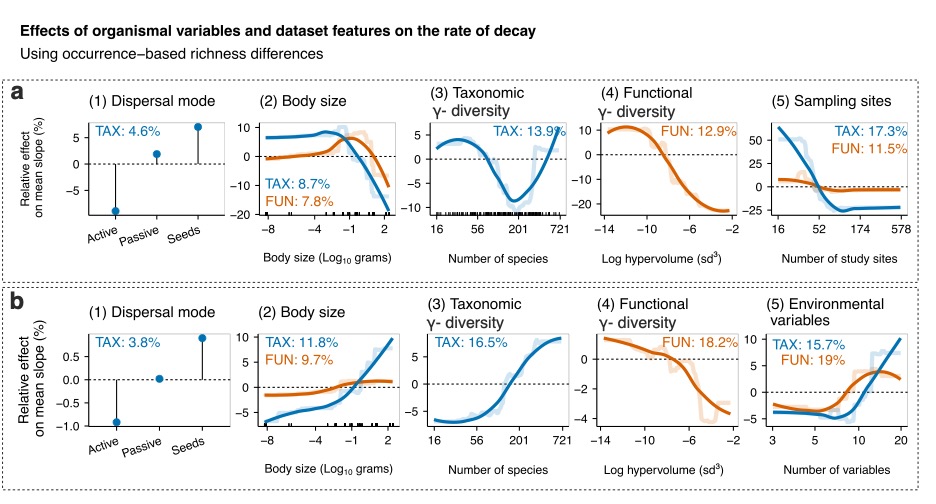


Figure S4.10. Relative effects (%) of organismal variables and dataset features on the rate of decay along spatial (a) and environmental (b) distance considering the richness differences component of taxonomic (blue lines) and functional (orange lines) similarities using occurrence data across datasets. Partial dependence plots show the effects of a predictor variable on the response variable after accounting for the average effects of all other variables in the model. Positive values indicate an increase in the rate of decay (steeper slopes) compared to the mean rate whereas negative values indicate a decrease in the rate of decay (flatter slopes) compared to the mean rate. Semi-transparent lines represent the actual predicted effects; solid lines represent LOESS fits to predicted values from BRT. We show here the organismal variables and the variables related to the dataset features.
