# Supplementary material for: Distance decay 2.0 – A global synthesis of taxonomic and functional turnover in ecological communities
Source: Glob Ecol Biogeogr. 2022 May 12;31(7):1399–421. doi: 10.1111/geb.13513 (PMC9322010; doi:10.1111/geb.13513)
Supplement: Supplementary file 5 — Appendix S5 [file GEB-31-1399-s002.docx]

Appendix to: Distance decay 2.0 – a global synthesis of taxonomic and functional decay in ecological communities

*Appendix S5*

**Additional analysis**

To investigate the possible drivers of the likelihood of taxonomic distance decay being stronger than functional distance decay, we used generalized linear models. We first constructed a binary variable where the value one was assigned to datasets with stronger taxonomic than functional distance decay (measured as Mantel r), and zero if otherwise. We included latitude, spatial extent, realms, body size, dispersal mode, γ-diversity, number of sites, and number of environmental variables as predictors, similarly to the Boosted Regression Trees. Models followed a Bernoulli distribution. We ran the models for environmental and spatial distance decays separately and included number of sites only in the GLM for spatial distance decay, while the number of environmental variables was included in the GLM for environmental distance decay.

Table 1. Summary of the generalized linear model following a Bernoulli distribution to test the variables affecting the likelihood of a dataset showing higher taxonomic than functional distance decay of similarities. The model was fitted using the difference between the Mantel R of the relationship between taxonomic and functional similarities along spatial distances as a response variable, where we assigned the “one” to datasets with higher taxonomic than functional Mantel R, and “zero” if otherwise.

| **R² = 0.23** | **Estimate** | **Standard Error** | **z-value** | **p-value** |
| --- | --- | --- | --- | --- |
| (Intercept) | -7.777 | 2.245 | -3.465 | **0.0005** |
| Latitude | 0.011 | 0.014 | 0.767 | 0.4433 |
| Spatial extent (Log) | 0.358 | 0.139 | 2.580 | **0.0099** |
| Marine vs. Terrestrial | 1.107 | 0.864 | 1.281 | 0.2003 |
| Freshwater vs. Terrestrial | 0.761 | 0.684 | 1.112 | 0.2661 |
| Body Size | -0.151 | 0.166 | -0.908 | 0.3638 |
| Passive vs. Active | -3.509 | 1.361 | -2.578 | **0.0099** |
| Seeds vs. Active | -0.049 | 0.625 | -0.078 | 0.9374 |
| Taxonomic γ-diversity (Log) | 0.714 | 0.299 | 2.390 | **0.0169** |
| Number of sites | 0.669 | 0.317 | 2.110 | **0.0348** |
| Functional γ-diversity (Log) | -0.160 | 0.079 | -2.023 | **0.0430** |
| (Dispersion parameter for binomial family taken to be 1) | | | | |
| Null deviance: 151.4 on 146 degrees of freedom | | | | |
| Residual deviance: 116 on 136 degrees of freedom | | | | |
| (1 observation deleted due to missingness) | | | | |

Table 2. Summary of the generalized linear model following a Bernoulli distribution to test the variables affecting the likelihood of a dataset showing higher taxonomic than functional distance decay of similarities. The model was fitted using the difference between the Mantel R of the relationship between taxonomic and functional similarities along environmental distances as a response variable, where we assigned the “one” to datasets with higher taxonomic than functional Mantel R, and “zero” if otherwise.

| **R² = 0.14** | **Estimate** | **Standard Error** | **z-value** | **p-value** | |
| --- | --- | --- | --- | --- | --- |
| (Intercept) | -5.680 | 1.897 | -2.994 | **0.0028** | |
| Latitude | 0.011 | 0.013 | 0.812 | 0.4165 | |
| Spatial extent (Log) | 0.278 | 0.131 | 2.127 | **0.0334** | |
| Marine vs. Terrestrial | 0.051 | 0.753 | 0.067 | 0.9464 | |
| Freshwater vs. Terrestrial | -0.177 | 0.615 | -0.288 | 0.7731 | |
| Body Size | 0.178 | 0.155 | 1.149 | 0.2504 | |
| Passive vs. Active | 0.435 | 1.213 | 0.359 | 0.7200 | |
| Seeds vs. Active | 0.581 | 0.614 | 0.945 | 0.3445 | |
| Taxonomic γ-diversity (Log) | 0.524 | 0.256 | 2.047 | **0.0407** | |
| Number of environmental variables | 1.230 | 0.445 | 2.763 | **0.0057** | |
| Functional γ-diversity (Log) | -0.034 | 0.070 | -0.482 | 0.6301 | |
| (Dispersion parameter for binomial family taken to be 1) | | | | |  |
| Null deviance: 154 on 146 degrees of freedom | | | | |  |
| Residual deviance: 132.1 on 136 degrees of freedom | | | | |  |
| (1 observation deleted due to missingness) | | | | |  |
